# High ploidy large cytoplasmic megakaryocytes are hematopoietic stem cells regulators and essential for platelet production

**DOI:** 10.1038/s41467-023-37780-7

**Published:** 2023-04-13

**Authors:** Shen Y. Heazlewood, Tanveer Ahmad, Benjamin Cao, Huimin Cao, Melanie Domingues, Xuan Sun, Chad K. Heazlewood, Songhui Li, Brenda Williams, Madeline Fulton, Jacinta F. White, Tom Nebl, Christian M. Nefzger, Jose M. Polo, Benjamin T. Kile, Felix Kraus, Michael T. Ryan, Yu B. Sun, Peter F. M. Choong, Sarah L. Ellis, Minna-Liisa Anko, Susan K. Nilsson

**Affiliations:** 1 Biomedical Manufacturing, Commonwealth Scientific and Industrial Research Organization, Melbourne, VIC Australia; 2 Australian Regenerative Medicine Institute, Monash University, Melbourne, VIC Australia; 3 Department of Anatomy and Developmental Biology, Monash University, Melbourne, VIC Australia; 4 Monash Biomedicine Discovery Institute, Melbourne, VIC Australia; 5 Department of Biochemistry and Molecular Biology, Monash University, Melbourne, VIC Australia; 6 Department of Surgery, St. Vincent’s Hospital, University of Melbourne, Melbourne, VIC Australia; 7 Bone and Soft Tissue Sarcoma Service, Peter MacCallum Cancer Centre, Melbourne, VIC Australia; 8 Department of Orthopaedics, St. Vincent’s Hospital Melbourne, Melbourne, VIC Australia; 9 Peter MacCallum Cancer Centre, Melbourne, VIC Australia; 10 The Sir Peter MacCallum Department of Oncology, The University of Melbourne, Melbourne, VIC Australia; 11 Centre for Reproductive Health and Centre for Cancer Research, Hudson Institute of Medical Research, Melbourne, VIC Australia; 12 Department of Molecular and Translational Science, Monash University, Melbourne, VIC Australia; 13 Present Address: Faculty of Medicine and Health Technology, Tampere University, Tampere, Finland

**Keywords:** Cell biology, Stem cells, Haematopoietic stem cells

## Abstract

Megakaryocytes (MK) generate platelets. Recently, we and others, have reported MK also regulate hematopoietic stem cells (HSC). Here we show high ploidy large cytoplasmic megakaryocytes (LCM) are critical negative regulators of HSC and critical for platelet formation. Using a mouse knockout model (*Pf4-Srsf3*^Δ/Δ^) with normal MK numbers, but essentially devoid of LCM, we demonstrate a pronounced increase in BM HSC concurrent with endogenous mobilization and extramedullary hematopoiesis. Severe thrombocytopenia is observed in animals with diminished LCM, although there is no change in MK ploidy distribution, uncoupling endoreduplication and platelet production. When HSC isolated from a microenvironment essentially devoid of LCM reconstitute hematopoiesis in lethally irradiated mice, the absence of LCM increases HSC in BM, blood and spleen, and the recapitulation of thrombocytopenia. In contrast, following a competitive transplant using minimal numbers of WT HSC together with HSC from a microenvironment with diminished LCM, sufficient WT HSC-generated LCM regulates a normal HSC pool and prevents thrombocytopenia. Importantly, LCM are conserved in humans.

## Abstract (web summary)

Not all megakaryocytes are created equal, with sub-populations identified that generate platelets and differentially regulate blood stem cells. These sub-populations, conserved in humans, are important in treating blood and clotting disorders.

## Introduction

Megakaryocytes (MK), known for platelet production, release factors including insulin-like growth factor-1 (IGF-1), insulin-like growth factor binding protein-3 (IGFBP-3), platelet factor-4 (PF4), thrombopoietin (TPO), transforming growth factor-β (TGF- β), factor X (FX) and prothrombin (PT) that regulate HSC^1–6^. We previously demonstrated that MK are randomly distributed in BM and ~75% of transplanted HSC (Lin^-^Sca1^+^c-Kit^+^CD150^+^CD48^-^) home to within two cell diameters of MK, where they are regulated by MK-released factors including IGF-1 and IGFBP-3^1^. Recently, Nakamura-Ishizu et al.^7^ demonstrated that ~70% of HSC reside within three cell diameters of MK, and HSC are induced into quiescence by MK-generated TPO during steady state. Similarly, MK-released PF4 and TGF-β were shown to induce HSC quiescence and MK ablation resulted in HSC expansion^2,4^. Furthermore, MK are important regulators of HSC through MK-released FX and PT, enabling thrombin-cleaved osteopontin (tcOPN) generation in situ; an important factor in HSC homing, retention and quiescence in BM via the integrins α_4_β_1_ (VLA-4) and α_9_β_1_^6,8,9^.

MK also indirectly regulate HSC by influencing the development of two well-known HSC regulators: osteoblasts (OB) and osteoclasts (OC). Post-irradiation, MK located near bone induce OB expansion via factors such as PDGF-BB, PDGF-β and bFGF; with this expansion in OB promoting HSC engraftment post-transplant^10,11^. MK also promote OB proliferation while inhibiting OC development through GATA-1, α_3_β_1_, α_5_β_1_, CD41 and Pyk2, resulting in an overall increase in bone formation^12–15^. In addition, in the calvaria, MK interact with osteomacs and OB to regulate HSC biology^16^. Recently, the sympathetic nervous system was shown to influence MK expansion post-myocardial infarction, demonstrating indirect regulation of HSC via the nervous system and MK^17–19^.

It has long been hypothesised that high ploidy MK generate platelets^20^. As MK mature, DNA replication occurs without cytokinesis, resulting in cells of increasing size and ploidy^20,21^. In mice and humans, the most common MK ploidy is 16N and it has been estimated that a single MK forms 3,000 platelets (reviewed in ref. ^22^). Many factors such as cyclin B1-dependent Cdc2 kinase, non-muscle myosin heavy chain IIB, guanine exchange factors GEF-H1 and ECT2, nicotinamide as well as effectors of Rho/Rock signalling are important in endoreplication (reviewed in ref. ^23^). Proplatelet formation and subsequent platelet release from MK occurs at the subluminal surface of sinusoidal endothelial cells; cytoplasmic protrusions of mature MK push through or between endothelial cells and are sheared off by blood flow (reviewed in ref. ^23^). Whether individual platelets are formed or larger cytoplasmic bodies are released and undergo further fragmentation is still being debated^24,25^. Although these events have been visualised in mouse calvaria^26^, the mechanisms initiating and regulating this process are not fully understood.

Herein we demonstrate MK of single ploidies can be sub-fractionated by antigen expression, such as CD41, into high ploidy large cytoplasmic MK (LCM) and high ploidy small cytoplasmic MK (SCM), and provide evidence that high ploidy LCM negatively regulate HSC function via factors including TPO and tcOPN. Using a mouse model, where the RNA binding protein, serine-arginine-rich splicing factor 3 (SRSF3)^27,28^, is deleted in MK and platelets using *Pf4*-*cre*^29^ (*Pf4-Srsf3*^Δ/Δ^ ^30^, herein termed MK-LCM^Δ/Δ^), normal MK numbers, but diminished LCM, elevated BM, PB and spleen HSC pools, as well as severe thrombocytopenia is observed. In addition, the negative HSC regulation is specific to the presence of BM LCM, evidenced through transplanting HSC isolated from a microenvironment with diminished LCM reconstituting the hematopoietic system, but recapitulating the diminished LCM phenotype, resulting in a dysregulated HSC pool as well as severe thrombocytopenia. However, minimal numbers of transplanted WT HSC were able to generate sufficient LCM to prevent the HSC defect and thrombocytopenia.

We also report the uncoupling of endoreduplication, with not all high ploidy MK being equivalent in ability to produce platelets, but high ploidy LCM being the primary source of platelets.

## Results

### Sub-fractionation of high ploidy large cytoplasmic MK (LCM) and high ploidy small cytoplasmic MK (SCM)

**Fig. 1:**
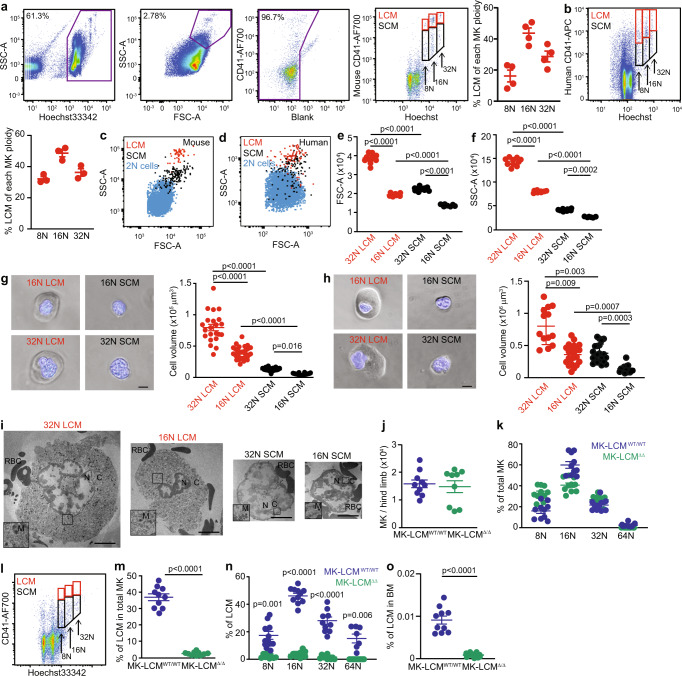
Sub-fractionation of high ploidy large cytoplasmic MK (LCM) and high ploidy small cytoplasmic MK (SCM). Sequential gating identifies high ploidy LCM and SCM: **a** ≥2N BM cells (purple gate); Large FSC^high^SSC^high^ cells; non-autofluorescent cells; MK ≥8N of individual ploidy further sub-fractionated using antigens such as CD41 into LCM (red gates, CD41^bright^) and SCM (black gates, CD41^dim^)(representative plots, *n* = 4, biological replicates from one experiment but this experiment has been performed ≥5 times). Back gating was performed to ensure high ploidy MK were captured within parental gates. 8N LCM = 0.0007 ± 0.0001%, 8N SCM = 0.0044 ± 0.0001% (total 8N = 0.0051%), 16N LCM = 0.0081 ± 0.0004%, 16N SCM = 0.0095 ± 0.0007% (total 16N = 0.018%), 32N LCM = 0.0018 ± 0.0003%, 32N SCM = 0.0048 ± 0.0005% (total 32N = 0.0066%) of total BM; average total high ploidy MK incidence in total BM = 0.0297%. **b** Human MK similarly separated into LCM and SCM (*n* = 3, biological replicates as independent experiments). 8N LCM = 0.03%, 8N SCM = 0.06% (total 8N = 0.09%), 16N LCM = 0.05%, 16N SCM = 0.05% (total 16N = 0.1%), 32N LCM = 0.015%, 32N SCM = 0.03% (total 32N = 0.045%). **c**, **d** LCM (mouse and human, respectively) exhibit higher FSC and SSC than SCM and 2N BM cells; each dot represents a cell (representative plots, *n* = 3, biological replicates, ≥3 independent experiments). **e** FSC of individual ploidy murine MK populations separated as LCM and SCM (*n* = 10, biological replicates from ≥2 independent experiments). **f** SSC of single ploidy murine MK populations separated as LCM and SCM (*n* = 10, biological replicates from ≥2 independent experiments). **g** Representative light microscopy images of prospectively isolated high ploidy murine LCM and SCM, cell volumes were calculated assuming all MK are spherical, blue = Hoechst 33342, scale bar = 10 μm, (32N LCM 22 cells, 16N LCM 37 cells, 32N SCM 43 cells, 16N SCM 40 cells all from *n* = 3, biological replicates from 3 independent experiments). **h** Images and cell volumes of prospectively isolated high ploidy human LCM and SCM (32N LCM 13 cells, 16N LCM 22 cells, 32N SCM 21 cells, 16N SCM 16 cells all from *n* = 2, biological replicates from 2 independent experiments). **i** Representative TEM images of prospectively isolated mouse LCM and SCM of single 16N ploidy. N = nucleus, C = cytoplasm, RBC = red blood cell, M = mitochondria. Scale bar = 5 μm (≥28 cells from *n* = 2, biological replicates and independent experiments). **j** Numbers of high ploidy MK in the hind limbs of MK-LCM^WT/WT^ (*n* = 10, biological replicates) and MK-LCM^Δ/Δ^ (*n* = 9, biological replicates) mice ≥2 independent experiments. **k** Ploidy distribution of high ploidy MK in MK-LCM^WT/WT^ and MK-LCM^Δ/Δ^ mice (*n* = 10, biological replicates, ≥2 independent experiments). **l** Representative flow cytometric plot of high ploidy MK-LCM^Δ/Δ^ MK separated on ploidy and sub-fractionated into LCM and SCM. 8N LCM = 0.0002 ± 0.0001%, 8N SCM = 0.0095 ± 0.0015% (total 8N = 0.0097%), 16N LCM = 0.0004 ± 0.0001%, 16N SCM = 0.0114 ± 0.0013% (total 16N = 0.0118%), 32N LCM = 0.0001 ± 0.0001%, 32N SCM = 0.0081 ± 0.0013% (total 32N = 0.0082%) of total BM; average total high ploidy MK incidence in total BM = 0.0297%, ≥5 independent experiments. **m** Frequency of high ploidy LCM in MK-LCM^WT/WT^ and MK-LCM^Δ/Δ^ mice (*n* = 10, biological replicates ≥2 independent experiments). **n** Frequency of high ploidy LCM in individual MK-LCM^WT/WT^ and MK-LCM^Δ/Δ^ MK ploidies (*n* = 10, biological replicates except 64N MK-LCM^WT/WT^, *n* = 6, biological replicates, ≥2 independent experiments). **o** Frequency of high ploidy LCM in the BM of MK-LCM^WT/WT^ and MK-LCM^Δ/Δ^ mice (*n* = 10, biological replicates, ≥2 independent experiments). Percentages shown in flow plots are current gate as a percentage of the parent. Statistical analysis used ordinary one-way ANOVA, *p* < 0.0001 (overall) with individual groups compared using Tukey’s multiple comparisons test, *p* values indicated for (**e**–**g**), Kruskal–Wallis test, *p* < 0.0001 (overall) with individual groups compared using Dunn’s multiple comparisons test, *p* values indicated for (**h**), unpaired two-sided Student’s *t*-test, *p* values indicated for (**m**, **o**), or two-way ANOVA with Geisser–Greenhouse correction, *p* < 0.0001 (overall) with individual groups for each timepoint compared using Holm–Sidak multiple comparisons test, *p* values indicated for (**n**). Source data are provided as a Source Data File. Error bars = SEM.

MK ploidy has routinely been accepted as a reflection of MK maturity^20^. We now demonstrate murine MK of individual ploidy can be sub-fractionated into LCM and SCM using antigens such as CD41 (Fig. 1a). Similarly, other antigens such as CD61, the binding partner of CD41, as well as c-Mpl can also sub-fractionate individual ploidy populations into LCM and SCM (Supplementary Fig. 1a, b). Importantly, the presence of LCM and SCM is replicated in human BM, with MK of individual ploidies showing distinct LCM and SCM populations (Fig. 1b).

Flow cytometric characterisation of murine and human LCM and SCM revealed unique forward scatter (FSC, an indication of size) and side scatter (SSC, an indication of complexity) profiles compared to other BM cells, with higher ploidy MK being significantly larger and more complex than lower ploidy MK, and LCM being significantly larger and more complex than SCM (Fig. 1c–f). This was further confirmed using light microscopy (Fig. 1g, h). TEM analysis of isolated murine MK demonstrated LCM have immense cytoplasm containing numerous platelet granules, from which proplatelet extensions and platelets form^31^ (Fig. 1i), suggesting a more mature MK subpopulation.

In addition to ploidy being reflective of MK maturity, the expression levels of transcription factors such as *Gata1/2*, *Nfe*2 and Z*fmp1* are key markers of MK maturation. Analysis of expression levels of these transcription factors in 16N SCM and LCM revealed increases in all four transcripts, although this was only significant for *Gata1* and Z*fmp1* (Supplementary Fig. 1c–f) further supporting this as the more mature MK subpopulation.

To better understand the functional difference between high ploidy LCM and SCM, we utilised a mouse model (Supplementary Fig. 1g, MK-LCM^Δ/Δ^*, Pf4-Srsf3*^Δ/Δ^mice) with normal incidence and total high ploidy MK numbers (≥8N, Fig. 1j). Within this high ploidy MK subpopulation the proportions of each MK ploidy are also equivalent to WT mice, but a non-significant trend of an increased proportion of 8N MK is observed (Fig. 1k). However, importantly this high ploidy MK subpopulation is essentially devoid of LCM (Fig. 1l–o and Supplementary Fig. 1h) which have been replaced with SCM (Supplementary Fig. 1i). Mice are viable, born according to the Mendelian ratio and similar to wildtype littermates and C57Bl/6 mice (MK-LCM^WT/WT^) in terms of weight, total BM cellularity and RBC count (Supplementary Fig. 1j–l). Consistent with SCM being a greater source of *Zfmp1* transcript than LCM, the BM of MK-LCM^Δ/Δ^ mice contained more ZFMP1 protein than LCM^WT/WT^ mice (Supplementary Fig. 1m).

### LCM regulate HSC quiescence

**Fig. 2:**
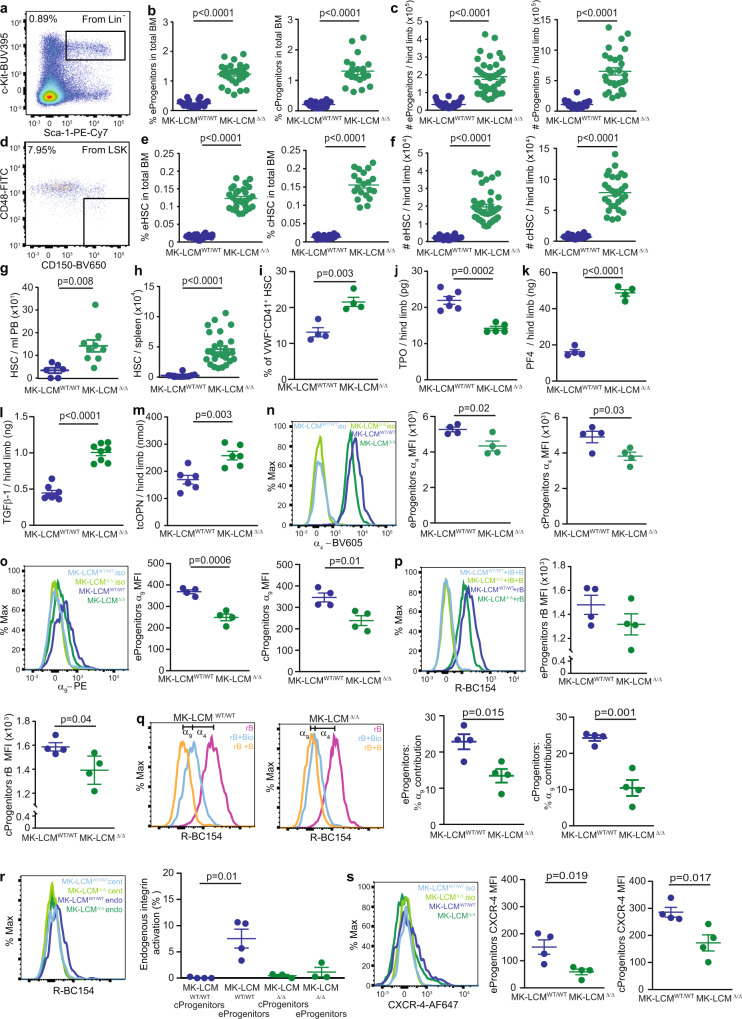
LCM regulate HSC quiescence. **a** Gating strategy for BM progenitors (LSK, Lin^-^Sca-1^+^c-Kit^+^, representative plot). **b** Frequency of LSK in endosteal (**e**) and central (**c**) MK-LCM^WT/WT^ and MK-LCM^Δ/Δ^ BM. **c** Absolute number of LSK in the eBM and cBM regions within a single hind limb. **d** Gating strategy for HSC: gating continued from **a** (Lin^-^Sca-1^+^c-Kit^+^CD150^+^CD48^-^, representative plot). **e** Frequency of MK-LCM^WT/WT^ and MK-LCM^Δ/Δ^ HSC in eBM and cBM. **f** Absolute number of HSC in eBM and cBM regions from one hind limb. **b**, **e** (eLCM^WT/WT^ *n* = 22, eMK-LCM^Δ/Δ^ *n* = 28, cLCM^WT/WT^ *n* = 16, cMK-LCM^Δ/Δ^ *n* = 20, biological replicates, from ≥5 independent experiments), **c**, **f** (eLCM^WT/WT^ *n* = 26, eMK-LCM^Δ/Δ^ *n* = 36, cLCM^WT/WT^ *n* = 20, cMK-LCM^Δ/Δ^ *n* = 28, biological replicates, from ≥5 independent experiments). **g** Absolute number of HSC in MK-LCM^WT/WT^ and MK-LCM^Δ/Δ^ PB (LCM^WT/WT^ *n* = 6, MK-LCM^Δ/Δ^ *n* = 9, biological replicates, from ≥3 independent experiments). **h** Number of HSC in MK-LCM^WT/WT^ and MK-LCM^Δ/Δ^ spleen (LCM^WT/WT^ *n* = 11, MK-LCM^Δ/Δ^ *n* = 26, biological replicates, from ≥4 independent experiments). **i** Frequency of VWF^+^CD41^+^ HSC in the BM of MK-LCM^WT/WT^ and MK-LCM^Δ/Δ^ mice (*n* = 4, biological replicates, from an experiment). **j** TPO concentration within the cBM fluid of MK-LCM^WT/WT^ and MK-LCM^Δ/Δ^ mice determined by ELISA (LCM^WT/WT^ *n* = 6, MK-LCM^Δ/Δ^ *n* = 5, biological replicates, from 2 independent experiments). **k** PF4 concentration within the cBM fluid of MK-LCM^WT/WT^ and MK-LCM^Δ/Δ^ mice as determined by ELISA (LCM^WT/WT^ *n* = 4, MK-LCM^Δ/Δ^ *n* = 4, biological replicates from 2 independent experiments). **l** ELISA data: TGFβ1 concentration within the cBM fluid of MK-LCM^WT/WT^ and MK-LCM^Δ/Δ^ mice (LCM^WT/WT^ *n* = 8, MK-LCM^Δ/Δ^ *n* = 8, biological replicates, from 2 independent experiments). **m** ELISA data: The concentration of tcOPN within MK-LCM^WT/WT^ and MK-LCM^Δ/Δ^ eBM lysates (LCM^WT/WT^ *n* = 6, MK-LCM^Δ/Δ^ *n* = 6, biological replicates, from 2 independent experiments). **n** Expression of α_4_ integrin on MK-LCM^WT/WT^ and MK-LCM^Δ/Δ^ e and c hematopoietic stem and progenitors. **o** Expression of α_9_ integrin on MK-LCM^WT/WT^ and MK-LCM^Δ/Δ^ e and c hematopoietic stem and progenitors. **p** Ability of MK-LCM^WT/WT^ and MK-LCM^Δ/Δ^ e and c hematopoietic stem and progenitors to bind R-BC154 (rB) and the ability of BOP (B) to outcomplete rB. **q** The contribution of α_9_ to rB binding on MK-LCM^WT/WT^ and MK-LCM^Δ/Δ^ e and c hematopoietic stem and progenitors, calculated based on rB binding, rB binding in the presence of the selective and potent α_4_β_1_ antagonist BIO5192 (Bio) and rB binding in the presence of B. **n**–**q** LCM^WT/WT^ *n* = 4, MK-LCM^Δ/Δ^ *n* = 4, biological replicates, from one experiment. **r** Degree of endogenous integrin activation on MK-LCM^WT/WT^ and MK-LCM^Δ/Δ^ **e** and **c** hematopoietic stem and progenitors (LCM^WT/WT^ *n* = 4, MK-LCM^Δ/Δ^ *n* = 3, biological replicates, from one experiment). **s** Expression of CXCR-4 on MK-LCM^WT/WT^ and MK-LCM^Δ/Δ^ **e** and **c** hematopoietic stem and progenitors (LCM^WT/WT^ *n* = 4, MK-LCM^Δ/Δ^ *n* = 4, biological replicates, from one experiment). Percentages shown in flow plots are current gate as a percentage of the parent. Statistical analysis used unpaired two-sided Student’s *t*-test, *p* values indicated for (**b**, **e**, **g**, **i**–**q**, **s**), two-sided Mann–Whitney test (**c**, **f**, **h**), Kruskal–Wallis test, *p* = 0.003 (overall) with individual groups compared using Dunn’s multiple comparisons test, *p* values indicated for (**r**). Source data are provided as a Source Data File. Error bars = SEM.

We, and others, previously showed that MK both directly and indirectly regulate BM HSC and progenitors^1,2,4–7^. In a microenvironment with diminished LCM, a significant increase in the frequency and absolute number of endosteal (e) and central (c) BM progenitors (Lin^-^Sca1^+^c-Kit^+^, Fig. 2a–c) and HSC (Lin^-^Sca1^+^c-Kit^+^CD150^+^CD48^-^, Fig. 2d–f) was observed, concurrent with endogenous mobilisation; with significantly increased numbers of progenitors and HSC in the PB (Fig. 2g and Supplementary Fig. 2a–d) as well as extramedullary hematopoiesis in the spleen (Fig. 2h and Supplementary Fig. 2e–h).

Somewhat surprisingly, cell cycle was not altered in HSC of mice with diminished LCM, (Supplementary Fig. 2i). In the absence of an increased total BM cellularity, the observation that mice with diminished LCM have an increased stem and progenitor cell pool that is not being correlated with an increase in cell cycle suggests these cells favour self-renewal over differentiation. Previously, this was a proposed mechanism for stem and progenitors devoid of MAD1 and p27^kip1^, where an expanded pool was also not associated with an increased cell cycle^32,33^. Although no difference in MK numbers was evident between MK-LCM^WT/WT^ or MK-LCM^Δ/Δ^ mice, increased ‘MK-primed’ VWF^+^CD41^+^ HSC were detected in the absence of LCM^34–36^ (Fig. 2i); however, whether this reflects a bias in HSC differentiation remains to be determined. A significantly increased basal respiration in MK-LCM^Δ/Δ^ HSC and progenitors was also evident, which is consistent with MK-primed HSC being more metabolically active^37^ (Supplementary Fig. 2j).

Importantly, the concentration of the MK-secreted protein TPO, known to play important roles in HSC and progenitor cell quiescence^7,38^ was significantly decreased in MK-LCM^Δ/Δ^ BM fluid (Fig. 2j). Interestingly, the concentration of TPO was elevated in MK-LCM^Δ/Δ^ mouse plasma (Supplementary Fig. 2k), supporting independent synthesis and regulation of TPO in BM and liver^5,7^. In addition, levels of the HSC quiescence-inducing cytokines PF4^39^ and TGFβ1^4^ were elevated in the BM fluid of MK-LCM^Δ/Δ^ mice (Fig. 2k, l and Supplementary Fig. 2l, m). Previously, fibroblast growth factor-1 (FGF-1) signalling has been demonstrated to transiently dominate over TGFβ quiescence to induce signalling to stimulate HSC expansion^4^; however, FGF-1 levels were actually significantly reduced in the BM of MK-LCM^Δ/Δ^ mice (Supplementary Fig. 2n).

Thrombin-cleaved OPN (tcOPN) has previously been demonstrated to be important for maintaining HSC and progenitor cell quiescence as well as adhesion to and retention within the BM microenvironment^8,9^, and MK contain the factors required to generate tcOPN within the BM microenvironment^6^. Somewhat surprisingly, reduced LCM did not correlate with reduced BM tcOPN, but actually resulted in significantly elevated tcOPN levels (Fig. 2m), suggesting LCM are not the dominant source of PT and FX; the factors required to generate tcOPN in situ. Furthermore, increased FX was evident in the BM microenvironment of MK-LCM^Δ/Δ^ (Supplementary Fig. 2o), explaining the increased tcOPN and suggesting SCM, not LCM, are the dominant source of BM FX. Consequently, HSC and progenitors isolated from a microenvironment with diminished LCM would not be as responsive to the quiescence-inducing cues of tcOPN due to significantly decreased expression of the receptors required to bind tcOPN: α_4_β_1_ (Fig. 2n) and α_9_β_1_ (Fig. 2o). Consistent with these results, HSC and progenitors isolated from MK-LCM^Δ/Δ^ mice had reduced capacity to bind R-BC154 (rB), a synthetic fluorescent analogue of the dual α_4_β_1_/α_9_β_1_ antagonist BOP^40^ (Fig. 2p). BOP (B), when in excess, outcompetes rB (Fig. 2p) and when the selective α_4_β_1_ antagonist, BIO5192 (Bio) is used in conjunction with rB and B, the individual contribution of α_4_β_1_ and α_9_β_1_ can be calculated^40^ (Fig. 2q). For rB and B to bind, integrin activation is required, which is mediated by the divalent metal cations Ca^2+^, Mg^2+^ and Mn^2^+^40^. As previously demonstrated, compared to centrally located progenitors, endosteally located progenitors are more strongly influenced by divalent metal cation gradients due to their close proximity to bone and as a result bind more rB without the addition of exogenous cations^40^ (Fig. 2r). However, endosteal MK-LCM^Δ/Δ^ progenitors showed a trend for reduced rB binding in the absence of exogenous cations, indicating reduced endogenous integrin activation despite their location. Collectively, this data suggests MK-LCM^Δ/Δ^ HSC and progenitors have decreased capacity to bind tcOPN both through reduced integrin expression, as well as decreased integrin activation, contributing to the HSC dysregulation and endogenous mobilisation.

MK-LCM^Δ/Δ^ HSC and progenitors also had significantly decreased CXCR-4 expression (Fig. 2s), with significantly decreased levels of SDF-1 evident in MK-LCM^Δ/Δ^ BM (Supplementary Fig. 2p), contributing to their release from the BM into the PB and spleen^41^. Furthermore, the protease MMP-9, which has previously been associated with HSC mobilisation^42,43^ was significantly increased in the BM of MK-LCM^Δ/Δ^ mice (Supplementary Fig. 2q, r). Together, the data provide evidence that LCM are important negative regulators of HSC and progenitors, and when LCM are essentially absent, HSC become dysregulated.

Other changes occurring in the BM microenvironment when no LCM are present include a decrease in IL-9, SCF and a large up-regulation of collagen VI α3 (Supplementary Fig. 2s–v). Collagen VI has been reported to increase human MSC proliferation^44^ and is a cytoadhesive element in the BM^45^. In addition, collagen VI gene modifications lead to platelet/clotting issues^46^. Collectively, these data indicate the absence of LCM has a profound and broad influence on the BM microenvironment, which in turn regulates HSC in a multi-faceted manner.

### HSC are regulated by LCM not platelet number

**Fig. 3:**
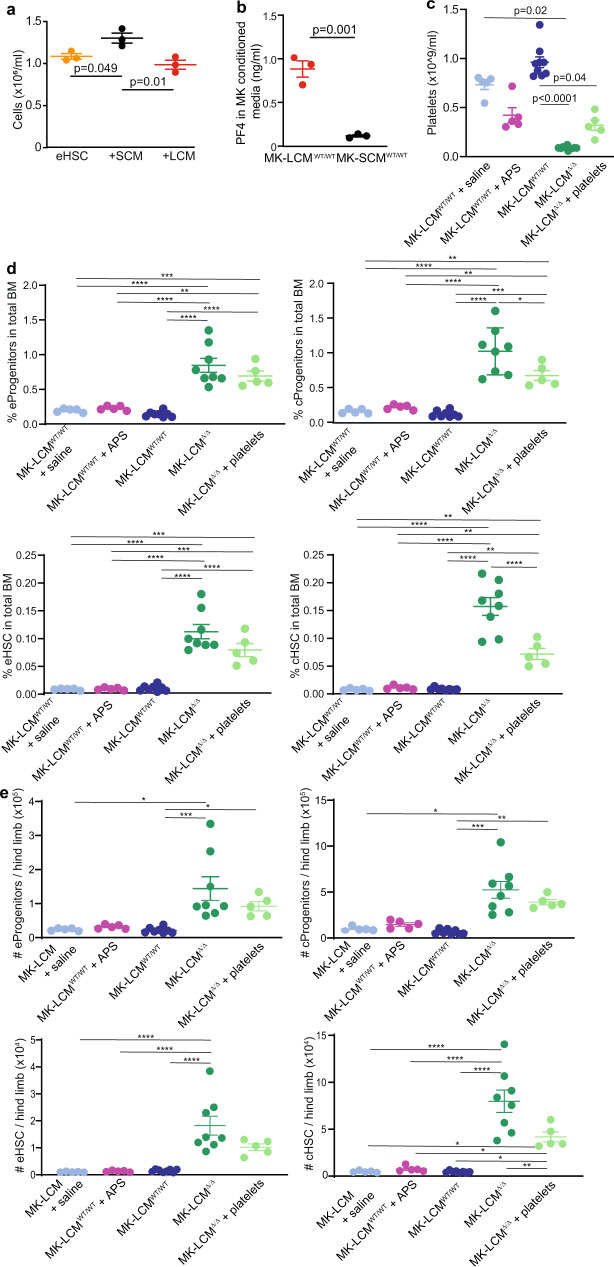
HSC are regulated by LCM not platelet number. **a** In vitro co-culture of endosteal (e) HSC with LCM or SCM (*n* = 3, biological replicate, one-way ANOVA *p* = 0.01, from one experiment). **b** PF4 released by MK-LCM^WT/WT^ LCM and MK-LCM^WT/WT^ SCM into culture media (*n* = 3, biological replicates, from one experiment, unpaired two-sided Student’s *t*-test). **c** Platelet counts following three doses of APS (+ APS) in MK-LCM^WT/WT^ mice, and repeated infusion of platelets (+ platelets) in MK-LCM^Δ/Δ^ mice compared to, MK-LCM^WT/WT^ + Saline (MK-LCM^WT/WT^ mice + APS diluent), MK-LCM^WT/WT^ and MK-LCM^Δ/Δ^ mice (MK-LCM^WT/WT^ + saline *n* = 5, MK-LCM^WT/WT^ + APS *n* = 5, MK-LCM^WT/WT^ *n* = 8, MK-LCM^Δ/Δ^ *n* = 8, MK-LCM^Δ/Δ^ + platelets *n* = 5, biological replicates, from one experiment), One-way ANOVA overall *p* < 0.0001, *p < 0.05, **p < 0.01, ***p < 0.001, ****p < 0.0001. **d** Frequency and **e** absolute number of progenitors and HSC in endosteal (**e**) and central (**c**) BM of mice from (**c**). Cell numbers are per hind limb (all one-way ANOVA overall *p* < 0.0001 except number of e and c progenitors and c HSC: Kruskal–Wallis *p* < 0.0001, *p < 0.05, **p < 0.01, ***p < 0.001, ****p < 0.0001). Source data are provided as a Source Data File. Error bars = SEM.

Importantly, co-culture assays demonstrated differential effects of LCM and SCM on proliferating HSC (Fig. 3a), with HSC co-cultured with SCM resulting in significantly increased cell proliferation and total numbers of progeny generated, while HSC co-cultured with LCM resulted in reduced total cell numbers post-culture. This is supportive of the in vivo data where mice with reduced LCM, but more SCM, have significantly increased haematopoietic stem and progenitors.

To better understand how LCM and SCM are functionally different, potentially altering their local microenvironment, their ability to release PF4 was assessed, revealing MK-LCM^WT/WT^ LCM release significantly more of the HSC quiescence-inducing factor than SCM (Fig. 3b); an observation that could be part of the mechanism resulting in the significant decrease in HSC proliferation evident in the presence of LCM compared to SCM (Fig. 3a). The factors released by SCM to significantly increase HSC proliferation compared to HSC alone warrant further investigation.

To determine if the altered HSC phenotype observed in MK-LCM^Δ/Δ^ mice is due to the concurrent thrombocytopenia, or the changes in LCM, we firstly repeatedly treated MK-LCM^WT/WT^ mice with anti-platelet serum (APS) to decrease their circulating platelet numbers to be equivalent to MK-LCM^Δ/Δ^ mice for a sustained period (Fig. 3c), then assessed the impact on the HSC and progenitor pool (Fig. 3d, e). No significant changes in the stem and progenitor pool were observed following an acute prolonged induction of thrombocytopenia, suggesting this alone is not the primary driver for the HSC phenotype in MK-LCM^Δ/Δ^ mice.

In addition, MK-LCM^WT/WT^ platelets were repeatedly infused into MK-LCM^Δ/Δ^ mice to boost circulating platelet numbers to within the normal range for a prolonged period (Fig. 3c) and the impact on the HSC and progenitor pool was assessed (Fig. 3d, e). Interestingly, the prolonged significant increase in platelet numbers resulted in a significant decrease in both the incidence and number of progenitors and HSC in the central BM compared to untreated MK-LCM^Δ/Δ^ mice. However, both the incidence and number of progenitors and HSC in these treated mice remained significantly higher than that observed in MK-LCM^WT/WT^ mice, further supporting thrombocytopenia alone is not the primary driver for the originally observed HSC phenotype.

### Dysregulation of the HSC pool is rescued by re-establishing LCM in BM

**Fig. 4:**
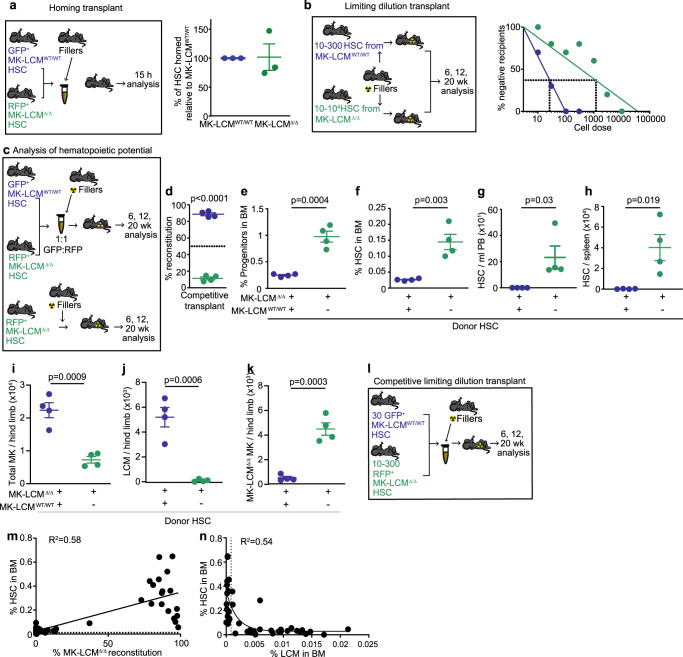
Dysregulated HSC pool is rescued by re-establishing BM LCM. **a** Homing of MK-LCM^WT/WT^ and MK-LCM^Δ/Δ^ HSC into the BM of C57Bl/6 mice 15 h post-transplant (*n* = 3, biological replicates, from one experiment). **b** Limiting dilution transplant outcome for mice receiving varying numbers of MK-LCM^WT/WT^ or MK-LCM^Δ/Δ^ HSC (*n* = 5 for MK-LCM^WT/WT^ cell doses of 100 and 300, *n* = 10 for MK-LCM^WT/WT^ cell doses of 10 and 30, *n* = 5 for MK-LCM^Δ/Δ^ cell doses of 10, 30, 1k, 3k and 10k and *n* = 10 for MK- LCM^Δ/Δ^ cell doses of 100 and 300, all biological replicates, from two experiments). **c** Schematic for the analysis of hematopoietic potential of GFP^+^MK-LCM^WT/WT^ plus RFP^+^MK-LCM^Δ/Δ^ or RFP^+^MK-LCM^Δ/Δ^ only HSC. **d** Percentage of donor contribution for a competitive transplant using equal numbers of MK-LCM^WT/WT^ plus MK-LCM^Δ/Δ^ HSC (blue), or MK-LCM^Δ/Δ^ HSC only (green). **e** Frequency of hematopoietic stem and progenitors and **f** HSC in mice reconstituted with MK-LCM^WT/WT^ plus MK-LCM^Δ/Δ^ or MK-LCM^Δ/Δ^ only HSC. **g** The number of HSC in the PB and spleen (**h**) following reconstitution with MK-LCM^WT/WT^ plus MK-LCM^Δ/Δ^ or MK-LCM^Δ/Δ^ only HSC. **i** Total MK number, **j** number of LCM and **k** number of MK of MK-LCM^Δ/Δ^ origin following reconstitution with MK-LCM^WT/WT^ plus MK-LCM^Δ/Δ^ or MK-LCM^Δ/Δ^ only HSC. **c**–**k** *n* = 4, biological replicates, from one experiment. **l** Schematic for competitive limiting dilution transplant (*n* = 5 per group, biological replicates). **m** Frequency of HSC in the BM relative to total BM MK-LCM^Δ/Δ^ reconstitution (*n* = 43 mice from 4 transplants, dotted line represents the frequency of HSC in MK-LCM^WT/WT^ BM). **n** Correlation between the frequency of HSC in BM as compared to the proportion of LCM in the BM of reconstituted mice (two-phase exponential decay, dotted line = 0.001%, *n* = 41 mice from 4 transplants). Statistical analysis used unpaired two-sided Student’s *t*-test, *p* values indicated for (**a**, **d**–**f**, **h**–**k)**, two-sided Mann–Whitney test (**g**), Poisson analysis (**b**), linear regression statistics (**m**), two-phase exponential decay (**n**). Source data are provided as a Source Data File. Error bars = SEM.

To determine if altering HSC regulation in MK-LCM^Δ/Δ^ mice is intrinsically or extrinsically regulated, HSC function was assessed by transplantation. Initially, the homing ability of HSC isolated from MK-LCM^WT/WT^ and MK-LCM^Δ/Δ^ mice was confirmed to be equivalent (Fig. 4a). Despite having a larger HSC pool, limiting dilution transplant analysis revealed HSC isolated from MK-LCM^Δ/Δ^ mice contained significantly fewer reconstituting HSC; 1 in 1190 (95% CI: 1 in 613–2311) versus 1 in 24 (95% CI: 1 in 18–33)(Fig. 4b). Consequently, it was not surprising when equal numbers of donor HSC were transplanted in a competitive transplant, MK-LCM^Δ/Δ^ HSC (RFP^+^) had significantly lower long-term engraftment potential compared to WT HSC (GFP^+^) (Fig. 4c, d). This is important, as even though there is a significant increase in phenotypic HSC in MK-LCM^Δ/Δ^ mice, they are not functional when assessed through transplant.

The extrinsic regulation of HSC by LCM was confirmed by the recapitulation of the original phenotype by a transplant of HSC from MK-LCM^Δ/Δ^ mice into lethally irradiated MK-LCM^WT/WT^ mice; evidenced by significantly increased HSC and progenitors in BM, PB and spleen (Fig. 4c, e–h). In contrast, when equivalent numbers of MK-LCM^WT/WT^ HSC were transplanted with HSC from MK-LCM^Δ/Δ^ mice, a normal stem and progenitor pool was evident in the BM and these cells were undetectable in the PB and spleen (Fig. 4c, e–h).

Following reconstitution with MK-LCM^WT/WT^ HSC plus MK-LCM^Δ/Δ^ HSC, significantly higher numbers of donor MK were evident compared to a reconstitution using MK-LCM^Δ/Δ^ HSC alone (Fig. 4i). In addition, the majority of MK in mice that received both MK-LCM^WT/WT^ and MK-LCM^Δ/Δ^ HSC were MK-LCM^WT/WT^ and MK numbers in these recipients were equivalent to steady-state MK-LCM^WT/WT^ mice (Fig. 4j and Supplementary Fig. 1h), demonstrating normal regulation of the HSC pool (Fig. 4e–h). Significant numbers of MK of MK-LCM^Δ/Δ^ phenotype were only evident when MK-LCM^Δ/Δ^ HSC were transplanted alone (Fig. 4k).

Furthermore, analysis of all the transplant conditions (Fig. 4b, c, l) suggests reconstitution with MK-LCM^Δ/Δ^ HSC recapitulates the MK-LCM^Δ/Δ^ phenotype evidenced by a dysregulated HSC pool (Fig. 4m), while reconstitution with MK-LCM^WT/WT^ HSC resulting in as few as 0.001% of LCM in whole BM (Fig. 4n, dotted line) rescues the MK-LCM^Δ/Δ^ phenotype, resulting in a normal sized HSC pool. The frequency of LCM resulting in a dysregulated HSC pool post-transplant is very similar to the frequency of LCM naturally occurring in the MK-LCM^Δ/Δ^ mice (Fig. 1o). Together the data demonstrate an extrinsic regulation of HSC, with LCM being critical regulators of HSC homoeostasis.

### *Pf4-Srsf3*^Δ/Δ^mice also have decreased lymphoid and increased myeloid cell frequencies in the BM, PB and spleen

In mice with diminished LCM, the frequency and absolute numbers of B- and T-cells are decreased in the BM (Supplementary Fig. 3a–d) while the frequency and absolute numbers of myeloid cells are increased in the BM (Supplementary Fig. 3e–f). In addition, the frequency of lymphoid cells is decreased (except for PB T-cells) and the frequency of myeloid cells is increased in the PB and spleen; however, due to the endogenous mobilisation and extramedullary hematopoiesis observed in these mice (Supplementary Fig. 2a, e), the absolute number of these populations are all increased (except for PB T-cells, where only a trend was observed)(Supplementary Fig. 3g–r, respectively).

### High ploidy LCM, and not SCM, generate platelets

**Fig. 5:**
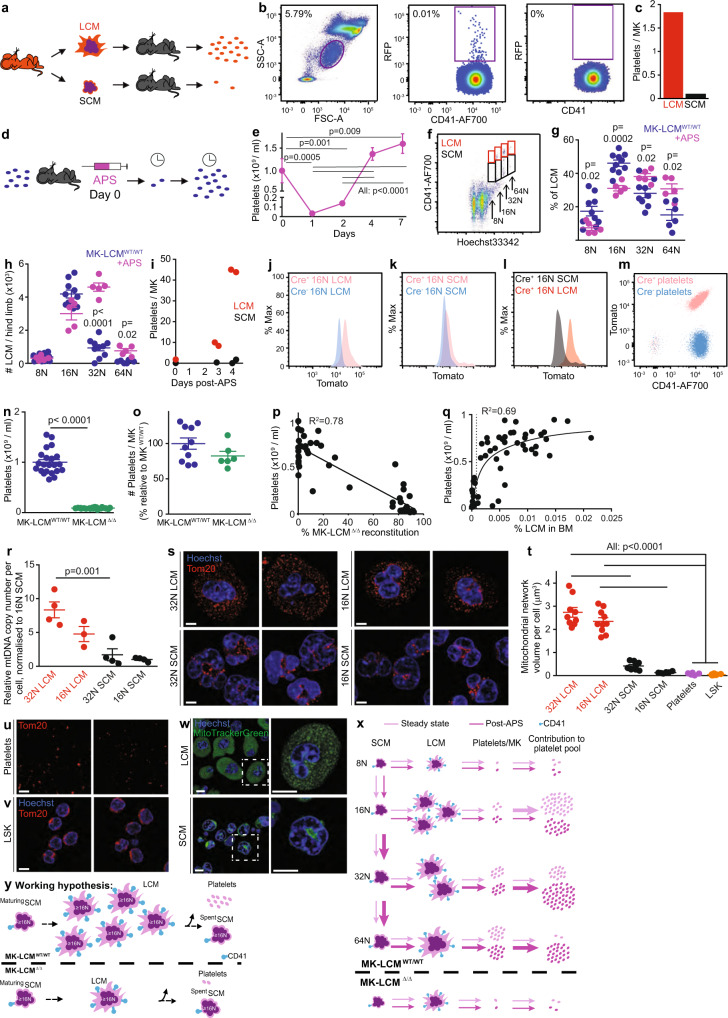
High ploidy LCM, and not SCM, generate platelets. **a** Schematic of MK transplant and in vivo platelet formation assay. **b** Platelets were identified by size and CD41 expression; transplanted RFP^+^ MK gave rise to RFP^+^platelets; non-inject control. **c** Number of platelets formed from each transplanted MK assuming all MK made equal numbers of platelets (*n* = 1). **d**, **e**) Schematic of platelet challenge and subsequent recovery following anti-platelet serum (APS) administration (*n* = 3, biological replicates, from one experiment). **f** Representative flow cytometry plot of BM MK during platelet recovery following an APS challenge (day 4). 8N LCM = 0.02%, 8N SCM = 0.26%, 16N LCM = 0.24%, 16N SCM = 0.57%, 32N LCM = 0.57%, 32N SCM = 0.65%, 64N LCM = 0.13%, 64N SCM = 0.18%. **g** Frequency and **h** absolute number of LCM within each ploidy ± APS treatment (day 4 data, APS *n* = 5, 64N *n* = 6, other ploidies *n* = 10, biological replicates, from one experiment). **i** RFP^+^ platelets in the PB of mice transplanted with RFP^+^ LCM or SCM following APS treatment, assuming all MK made equal numbers of platelets (day 0 *n* = 1, day 3 *n* = 1 for SCM and 2 for LCM, day 4 *n* = 2, biological replicates, from two experiments). **j** Representative data: tdTomato expression in LCM and **k** SCM of *Pf4-Cre* driven tdTomato expressing mice versus control mice. **l** tdTomato expression in the LCM and SCM of *Pf4-Cre*^*+*^ tdTomato expressing mice. **m** tdTomato expression in platelets of *Pf4-Cre* driven tdTomato expressing mice versus controls (**j**–**m**: *n* = 9, biological replicates, from two experiments). **n** Platelet number in MK-LCM^WT/WT^ and MK-LCM^Δ/Δ^ PB (MK-LCM^WT/WT^ *n* = 22, MK-LCM^Δ/Δ^ *n* = 20, biological replicates, from eight experiments). **o** Number of platelets generated per MK if only LCM generate platelets. Data presented were normalised to MK-LCM^WT/WT^ mice and assumed a 20 g mouse contained 1.5 ml of PB^94^, MK are randomly distributed in BM^1^ and BM from one hind leg accounts for 15% of total BM^95^ (MK-LCM^WT/WT^ *n* = 10, MK-LCM^Δ/Δ^ *n* = 6, biological replicates, from three experiments). **p** The relationship between MK-LCM^Δ/Δ^ reconstitution in the PB and platelet counts (*n* = 55, biological replicates, from four transplants). **q** Platelet counts in transplanted mice relative to the proportion of LCM in the BM (exponential 2-phase association, dotted line = 0.001%, *n* = 54, biological replicates, from four transplants). **r** RT-PCR analysis of MK mitochondrial and genomic DNA (All *n* = 4 except 16N LCM *n* = 3, biological replicates, from two experiments). **s** Representative Tom20 staining of prospectively isolated MK (2 independent experiments were performed). **t** Quantification of mitochondrial networks in MK, platelets and HSC (32N LCM *n* = 9 cells, 16N LCM *n* = 10 cells, 32N SCM *n* = 9 cells, 16N SCM *n* = 7 cells, platelets *n* = 9, LSK *n* = 10 cells). **u** Representative Tom20 staining in platelets and **v** LSK cells (2 independent experiments were performed). **w** Representative MitoTrackerGreen staining of sorted MK (2 independent experiments were performed). **x** Hypothesised platelet release and contribution of single ploidy MK under haemostatic conditions and following the induction of acute thrombocytopenia. **y** Summary hypothesis of MK maturing from ^Maturing^SCM to LCM then ^Spent^SCM following platelet release. Scale bar = 5 μm except (**w**) inset = 10 μm, percentages are of the parent. Statistical analysis used ordinary one-way ANOVA, *p* < 0.0001 (overall) with individual groups compared using Tukey’s multiple comparisons test, *p* values as shown (**e**), two-way ANOVA with Geisser–Greenhouse correction, *p* < 0.0001 (overall) with individual groups compared using Holm–Sidak multiple comparisons test, *p* values indicated for (**g**), two-way ANOVA with Geisser–Greenhouse correction, *p* = 0.0004 (overall) with individual groups compared using Holm–Sidak multiple comparisons test, *p* values indicated for (**h**), unpaired two-sided Student’s *t*-test, *p* values indicated for (**n**–**o**), linear regression statistics (**p**) and two-phase exponential association curve (**q**), ordinary one-way ANOVA, *p* = 0.0004 (overall) with individual groups compared using Tukey’s multiple comparisons test, *p* values indicated for (**r**, **t**). Source data are provided as a Source Data File. Error bars = SEM.

While it is widely accepted that high ploidy MK generate platelets, to investigate functional differences between high ploidy LCM and SCM, cells were isolated from red fluorescent protein (RFP) mice and transplanted into C57Bl/6 mice (Fig. 5a) and their ability to generate RFP^+^ platelets 90 min post-transplant assessed (Fig. 5b). High ploidy (≥16N) LCM generated ~18.5-fold more platelets than high ploidy (≥16N) SCM (Fig. 5c).

APS is routinely used to deplete circulating platelets in mice with platelet recovery occurring within a week (Fig. 5d, e), which we hypothesised to be driven by high ploidy LCM. Indeed, the frequency and number of total MK was significantly increased (~50%) 4 days post-APS, with an increase in MK of higher ploidy, but more importantly, a significant shift in the proportion of LCM in individual ploidies, resulting in a 5.6-fold and an 8.4-fold increase in the number of 32N and 64N LCM, respectively (Fig. 5f–h). The ability of these newly generated high ploidy LCM (≥16N) to rapidly generate platelets was confirmed by transplant, with APS-primed RFP^+^ ≥16N LCM generating 24.3-fold and 43.9-fold more platelets compared to steady state ≥16N LCM and APS-primed RFP^+^ ≥16N SCM respectively (Fig. 5i).

The ploidy distribution and frequency of LCM in mice where *Pf4* drove the expression of tdTomato was equivalent to controls (*Pf4-Cre Stop*^*fl/fl*^*tdTomato*, Supplementary Fig. 4a, b), with tdTomato expression in *Pf4-Cre*^*+*^ LCM confirmed (Fig. 5j). The expression of tdTomato in SCM was similar between Cre^+^ and Cre^-^ mice, with a small but distinct subset of Cre^+^ SCM with high tdTomato expression (Fig. 5k). Within *Pf4-Cre Stop*^*fl/fl*^*tdTomato* Cre^+^ mice, LCM highly expressed tdTomato while the majority of SCM did not (Fig. 5l). Furthermore, the >99% of platelets from *Pf4-Cre Stop*^*fl/fl*^*tdTomato* Cre^+^ mice were tdTomato^+^ (Fig. 5m), further supporting our hypothesis that LCM, and not SCM, are the main source of platelets.

The importance of LCM in platelet generation was further confirmed in MK-LCM^Δ/Δ^ mice; the ~90% reduction in LCM (Fig. 1m) coincided with a ~90% reduction in platelets (Fig. 5n). Nevertheless, assuming LCM are the dominant source of platelets as described above, the very few residual LCM present in MK-LCM^Δ/Δ^ mice were capable of generating as many platelets as LCM from MK-LCM^WT/WT^ mice (Fig. 5o), but due to their low frequency, MK-LCM^Δ/Δ^ mice are severely thrombocytopenic. Following transplant and reconstitution with MK-LCM^Δ/Δ^ HSC (Fig. 4b, c, l), mice with high MK-LCM^Δ/Δ^ chimerism recapitulated the original phenotype of severe thrombocytopenia, while mice with low MK-LCM^Δ/Δ^ HSC reconstitution had normal platelet counts (Fig. 5p). Overall, the frequency of LCM in the BM directly correlated with platelet number; when the frequency of LCM was less than 0.001% of total BM, mice were thrombocytopenic (dotted line, Fig. 5q) and where transplanted MK-LCM^WT/WT^ HSC generated sufficient LCM (>0.006% in total BM), mice had normal platelet counts.

Since increased mitochondrial biogenesis has been associated with MK maturation and platelet production in culture^47^, we hypothesised LCM would contain more mitochondria than SCM. Indeed, RT-PCR revealed LCM contained more mitochondrial DNA (Fig. 5r) and quantification of confocal images of prospectively isolated MK stained with an anti-mitochondrial import receptor (Tom20) demonstrated significantly more mitochondria in LCM compared to SCM (Fig. 5s, t). Mitochondria in LCM and SCM were fragmented and packaged into platelets in this form (Fig. 5u), which is in contrast to normal, where mitochondria undergo constant fusion and fission and have a reticular morphology as demonstrated in LSK cells (Fig. 5v). While mitochondria in LCM and SCM were demonstrated to be functional using MitoTrackerGreen staining (Fig. 5w), due to the low frequency of LCM and SCM in BM, measurements of oxygen consumption were not possible using the Seahorse assay. Although the functional significance of fragmented mitochondria in MK remains unclear, these data support high ploidy LCM being the more mature, platelet-producing MK (Fig. 5x, y).

## Discussion

**Fig. 6:**
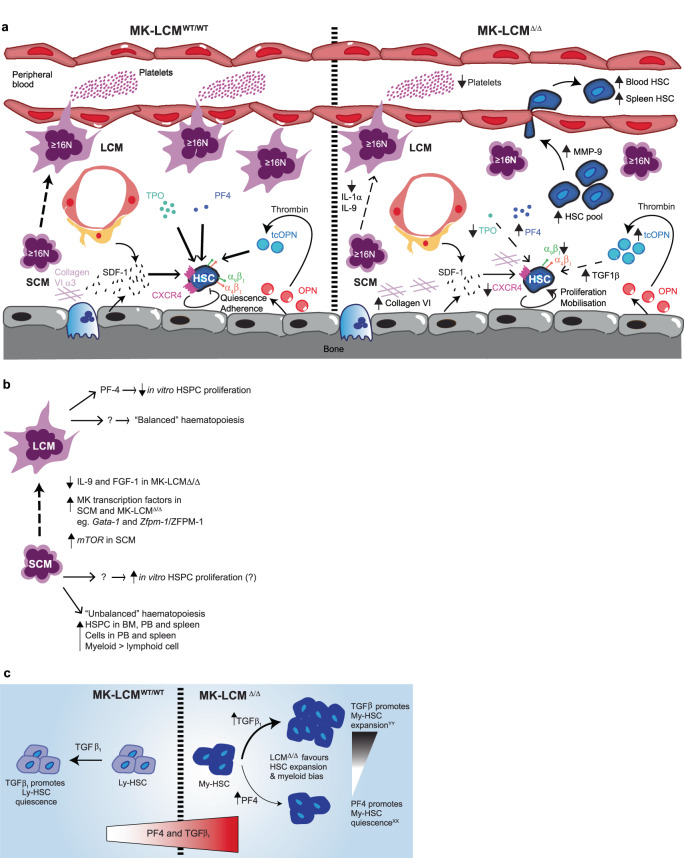
Models of MK:HSC interactions in BM. **a** Schematic depicting several mechanisms by which a loss of BM LCM results in increased BM hematopoietic stem and progenitors, endogenous mobilisation and splenomegaly. **b** Schematic depicting how LCM and SCM could differentially regulate HSC. **c** Schematic depicting how TGFβ1 and PF4 could be involved in these observations.

Although MK are randomly distributed in BM, HSC preferentially home near MK post-transplant and influence HSC proliferation in a non-contact dependent manner (previously, the mechanism for this has been demonstrated to include the release of cytokines such as IGFBP-3 and IGF-1), highlighting MK as an important component of the HSC niche^1,48^. We now demonstrate that even with physiologically normal total numbers of high ploidy MK, the absence of high ploidy LCM and concurrent significant increase in high ploidy SCM, results in HSC dysregulation and severe thrombocytopenia; identifying LCM as key negative regulators of HSC and essential for platelet production and SCM as key positive regulators of HSC, but unable to produce physiologically normal numbers of platelets (Fig. 6a).

Importantly, functional analysis demonstrates LCM and SCM are different in their ability to regulate HSC proliferation, with co-culturing SCM with HSC in vitro resulting in a significant increase in the total number of HSC-derived progeny, which is similar to that evident in our mouse model characterised by a significant increase in SCM in the almost complete absence of LCM. In addition, we demonstrate LCM release significantly more PF4 than SCM, identifying a likely mechanism in the regulation of HSC, with previous studies identifying MK-secreted PF4 as a critical negative regulator of HSC^2^.

In addition, we recently used RNA-sequencing analysis of MK isolated from MK-LCM^Δ/Δ^ and MK-LCM^WT/WT^ mice to demonstrate SRSF3 is critical during MK maturation^30^. Further analysis now reveals the serine/threonine protein kinase *mTOR* is significantly downregulated (2.3-fold) as WT 8N MK transition to 16N, but this significant decrease is absent in the transition of MK-LCM^Δ/Δ^ 8N MK to 16N, with *mTOR* levels remaining unchanged and equivalent to WT 8N MK. Importantly, the absence of the observed decrease in *mTOR* expression as MK mature from 8N to 16N in MK-LCM^Δ/Δ^ mice, that are devoid of LCM, coinciding with a significant increase in LCM as WT MK mature from 8N to 16N also suggests *mTOR* is more prevalent in SCM compared to LCM. Together this suggests a role for *mTOR* in MK maturation and potentially for SCM to mature to LCM, with previous studies demonstrating mTOR is critical in driving protein synthesis, mitochondrial function and cell size^49–51^ including MK ploidy and cell size^52^ (Fig. 6b).

However, these changes in mTOR are unlikely to be part of the direct mechanism through which MK are regulating HSC. As mentioned above, PF4 is a critical negative regulator of HSC^2^, but in our MK-LCM^Δ/Δ^ mouse model, we saw both a significant increase in PF4, and a concurrent significant increase in HSC proliferation. Contention still remains as to whether HSC express PF4, with studies reporting both minimal expression in mature hematopoietic cells in the BM, spleen and thymus, or up to 53.2% of HPC and 40.5% of HSC expressing Pf4-Cre^29,53^. Using the *Pf4* promoter to drive tdTomato expression^53^ (*Pf4-Cre-tdTomato*), we observed only 6.5 ± 0.7% of HSC and 2.3 ± 0.2% of HSPC express tdTomato, strongly suggesting MK-LCM^Δ/Δ^ HSC themselves are not responsible for the phenotype evident in the mouse model, as tdTomato^+^ HSC do not proliferate to become dominant in the HSC pool. In addition, following transplants of a pool of MK-LCM^Δ/Δ^ and MK-LCM^WT/WT^ HSC, the MK-LCM^Δ/Δ^ HSC phenotype is not recapitulated, demonstrating the phenotype is not cell intrinsic.

Alternatively, the increased PF4 could, in part, be due to the presence of activated platelets in MK-LCM^Δ/Δ^ mice^30^, although total platelet numbers are very low in these animals. Interestingly, MK-LCM^Δ/Δ^ mice also present with a significantly increased proportion of BM myeloid cells and whilst platelets are known as a dominant source of PF4, monocytes also contain and release significant amounts of PF4^54^. Increased BM PF4 in MK-LCM^Δ/Δ^ mice could be involved in the observed increased generation of VWF^+^CD41^+^ (MK-primed) HSC^36^. Previously, the depletion of MK has been shown to expand MK-biased HSC with reduced transplant potential^36^. However, the current data suggests this regulation may not be due to generic MK, but LCM, or the lack of LCM in the BM microenvironment.

The mechanism underpinning this significantly increased frequency of myeloid cells in the BM, PB and spleen evident in MK-LCM^Δ/Δ^ mice is likely to involve TGFβ1, which is known to drive myeloid biased HSC proliferation^55,56^ and is also significantly increased in MK-LCM^Δ/Δ^ mice (Fig. 6c). However, like PF4, TGFβ1 has also been reported to induce HSC quiescence^4,39^, whereas we observed an enlarged HSC pool. These previously identified mechanisms of HSC regulation may be overshadowed by other mechanisms such as thrombopoietin (TPO).

Thrombopoietin derived from stromal cells, osteoblastic cells and MK also induces quiescence in HSC^7,38,57,58^ and HSC in TPO KO mice have accelerated cycling leading to HSC exhaustion^59^. Furthermore, HSC and progenitors starved of TPO have increased proliferation and exhaust with age^60^. Significantly reduced TPO was evident in MK-LCM^Δ/Δ^ mouse BM fluid, providing an additional mechanism for the dysregulated HSC pool, and supporting in situ BM TPO regulation^5,7^. However, differential TPO mRNA expression was not detected in LCM and SCM, supporting liver-generated TPO as important for HSC maintenance^61^. Regardless, as reduced TPO levels in the BM of MK- LCM^Δ/Δ^ mice were correlated with significantly increased levels in the blood, it is evident that the amount of TPO in the local BM microenvironment is important for HSC regulation, and therefore, we hypothesise injecting TPO into MK-LCM^Δ/Δ^ mice will not increase BM levels and rescue the HSC dysfunction evident in these mice.

Endosteal BM tcOPN is also known to induce HSC quiescence and decrease myeloid commitment through interactions with α_4_β_1_ and α_9_β_1_ integrins^8,9^. Interestingly, MK-LCM^Δ/Δ^ mice have increased amounts of endosteal BM tcOPN but a significantly larger HSC pool and increased myeloid commitment. However, in the absence of LCM in this model, HSC and progenitors express significantly decreased levels of α_4_β_1_ and α_9_β_1_ integrins, and significantly fewer of these receptors are endogenously in an activated confirmation. Together, this results in a decreased responsiveness to the quiescence-inducing cue of tcOPN, as well as an increase in myeloid differentiation.

In addition, endogenous mobilisation is also evident in MK-LCM^Δ/Δ^ mice and since the retention of HSC and progenitors within BM is, at least in part, regulated by tcOPN^9^, the decreased responsiveness of MK-LCM^Δ/Δ^ HSC and progenitors to tcOPN is likely to play a role in their migration to the PB and spleen. This is supported by previous observations demonstrating that inhibiting α_4_β_1_ or α_9_β_1_ integrins, or the CXCR-4/SDF-1 interactions, induces HSC and progenitor cell release from BM^40,41^ (Fig. 6a). Furthermore, the increased MMP-9 evident in the BM of MK-LCM^Δ/Δ^ mice could be involved in the degradation of HSC adhesion molecules or extracellular matrix/basement membrane^42,43,62^, allowing for the detachment of HSC from the BM niche and contributing to the endogenous mobilisation evident in these mice. In addition, although SDF-1 is known to influence HSC quiescence, differentiation as well as migration^63^, the significantly reduced expression of CXCR-4 on the surface MK-LCM^Δ/Δ^ HSC would result in a decreased responsiveness to SDF-1.

Diminished LCM numbers and an enlarged HSC and progenitor cell pool were recapitulated when transplanted MK-LCM^Δ/Δ^ HSC reconstituted hematopoiesis. However, following a competitive transplant, even low MK-LCM^WT/WT^ HSC reconstitution resulted in the production of normal numbers of WT LCM, stabilising the HSC pool as well as resulting in the absence of endogenous and extramedullary haematopoiesis; demonstrating the key regulatory role of LCM and dysregulation in their absence.

Importantly, even though restoring platelet numbers in MK-LCM^Δ/Δ^ mice resulted in a significant decrease in the incidence and number of HSC, both the incidence and number of HSC remained significantly higher than in MK-LCM^WT/WT^ mice. Together with an acute prolonged induction in thrombocytopenia also having no impact on the stem and progenitor pool, suggests thrombocytopenia alone is not the primary driver for the originally observed HSC phenotype in MK-LCM^Δ/Δ^ mice. These findings are supported by Bruns et al. who demonstrated Neuraminidase treatment (which similarly reduces platelet number without affecting MK numbers), also had no impact on the HSC pool, ‘suggesting that MK, but not platelets, regulate BM HSC quiescence’^64^.

The data demonstrate that both SCM and LCM are important regulators of HSC and progenitors, but LCM are also responsible for platelet formation. The presence and functional significance of LCM sub-populations was previously unappreciated in human BM^65^. A protocol for the isolation and in vivo assay of LCM and SCM function is now provided. Following the induction of acute thrombocytopenia, isolated and transplanted LCM released ~44.5 times more platelets than SCM, and ~24.5 times more platelets than LCM isolated from non-thrombocytopenic mice; suggesting LCM in a thrombocytopenic environment are primed to make platelets, with no differences in forward and side scatter of APS-primed LCM and SCM detected compared to non-APS induced MK. The proportion of platelets generated by MK of specific ploidy was originally estimated as 5.6% from 8N, 55% from 16N and 39% from 32N MK^66,67^. Following APS, 32N and 64N LCM become the dominant sub-populations responsible for platelet generation (Fig. 5x). Furthermore, a ~90% reduction in LCM is accompanied by a ~90% reduction in platelets, but the extremely rare LCM individually generate equivalent numbers of platelets to MK-LCM^WT/WT^.

Historically, SCM may have been classified as ‘naked nuclei’ or MK with ‘disintegrated cytoplasms’ and attributed to technical processing issues^68^. We hypothesise SCM are an important subset of MK that can be further subsetted into maturing SCM that will become LCM (^Maturing^SCM), making up the vast majority of SCM, but not yet express PF4 and therefore do not express tomato in *Pf4-Cre Stop*^*fl/fl*^*tdTomato* mice (Fig. 5l, y), and a small but distinct population of LCM that have released their platelets and are ^Spent^SCM that express PF4 and therefore tomato in *Pf4-Cre Stop*^*fl/fl*^*tdTomato* mice (Fig. 5l, y) that would be removed from the BM via phagocytosis by macrophages^69^. We hypothesise MK maturation factors such as IL-9 and FGF-1, which are both significantly reduced in MK-LCM^Δ/Δ^ mice, are important for the SCM to LCM transition^70,71^.

The concept of MK requiring significant cytoplasm to generate platelets was proposed by Kaufman et al. in 1965^68^. The current data support the notion that ploidy maturation in MK precedes cytoplasmic maturation^72,73^ with no defect in high ploidy MK generation observed in MK-LCM^Δ/Δ^ mice, only the formation of LCM. This is further evidenced by increased MK-primed VWF^+^CD41^+^ HSC in MK-LCM^Δ/Δ^ mice. No change in MK number or ploidy distribution was observed in mice lacking LCM, divorcing MK generation and ploidy maturation from the generation of platelet forming, high ploidy LCM.

Previous data suggest 8N MK generate larger, denser platelets, while 32N MK generate lighter platelets with fewer granules and mitochondria^66,67^. In the current study, no significant difference in the mitochondrial networks of 32N LCM and 16N LCM was evident, but both populations had significantly larger mitochondrial networks than 32N SCM, 16N SCM or HSC, despite the mitochondria being fragmented. In addition, previous analyses of cell lines and cultured MK suggests MK are metabolically active, with mitochondria, as an intracellular source of energy and reactive oxygen species, being important for MK maturation, including the processes of polyploidation and proplatelet formation^47,74,75^. The current data suggest that fragmentation is important for packaging mitochondria into platelets.

Importantly, whilst CD41 can be used to routinely isolate LCM and SCM, these cells are identified by the amount of cytoplasm individual MK possess, not the expression of CD41 per se, with other antigens such as CD61 and c-MPL also sub-fractionating LCM and SCM. Consequently, CD41 KO animals would be expected to contain similar numbers of LCM and SCM. Indeed, no impact on platelet numbers was detected following the knocking-out of CD41^76^.

While low platelet counts (due to disease or induced thrombocytopenia) in patients and animal models commonly increase MK frequency and ploidy, MK maturation as determined by ploidy alone, is not an accurate indicator of thrombocytosis (reviewed in ref. ^23^). In many cases of chronic granulocytic leukaemia and reactive thrombocytosis, patients present with MK of lower ploidy but have normal or even higher than average platelet counts, while patients with idiopathic/immune thrombocytopenic purpura have fewer platelets generated from MK of high ploidy^77,78^. Numbers of MK in patients with Wiskott–Aldrich syndrome (WAS) are considered normal, or higher than normal, but thrombocytopenia is evident (reviewed in ref. ^79^). WAS has been suggested to be caused by increased circulating anti-platelet antibodies leading to increased clearance^80,81^ but since WASp knockout mice have increased ‘naked’ MK^82^, the possibility that WAS patients have decreased LCM and increased SCM, giving patients ‘normal’ MK numbers, but thrombocytopenia should be explored. Therefore, assessing LCM and SCM frequency, and not just ploidy or total MK numbers, should give more insight into thrombocytopenias. Furthermore, although analysis of platelet survival and function in MK-LCM^Δ/Δ^ mice is beyond the scope of the current study, reduced platelet counts could also be, at least in part, explained by impaired survival, with platelets from these mice known to be cleared faster^30^.

In summary, the data show high ploidy LCM are important negative regulators of HSC and essential for platelet production and SCM as key positive regulators of HSC, but unable to produce physiologically normal numbers of platelets and ploidy alone does not determine the ability of MK to generate platelets. In mice with diminished LCM, the HSC pool becomes enlarged and both endogenous mobilisation and extramedullary hematopoiesis are evident. Prospectively isolated LCM, but not SCM, form platelets in vivo and these MK sub-fractions are conserved in humans. Therefore, sub-fractioning MK as LCM and SCM, in isolation or within individual ploidies, would be a better predictor of MK platelet-generating potential and more accurately reflect a patient’s platelet count. Furthermore, therapeutic efforts to increase platelet production in vitro or in vivo by increasing total MK (or increasing MK of higher ploidy), must also specifically increase the fraction of LCM. However, careful consideration must also be given to the potential consequences of LCM being negative regulators of HSC, and altering the frequency and number of LCM having an impact on steady-state hematopoiesis.

## Methods

### Mice

C57Bl/6 (MK-LCM^WT/WT^) mice were purchased from the Monash Animal Research Platform (MARP, Monash University, Australia). Mice carrying the floxed serine-arginine rich splicing factor 3 (*Srsf3* ^*fl/fl*^) conditional allele^83^ were crossed with C57BL/6-Tg(Pf4-icre)Q3Rsko/J mice to generate platelet factor-4 (*Pf4*)-*Cre*-*Srsf3* KO (MK-LCM^Δ/Δ^) mice, where deletion of exon 2 and 3 of *Srsf3* was under the control of the *Pf4* promoter. *Pf4*-*Cre*-*Srsf3* KO mice were bred with RFP mice ubiquitously expressing a cytoplasmic pbActin-CMV-DsRed T3 transgene to generate RFP *Pf4-Cre*-*Srsf3* KO mice. *Pf4-Cre* mice were separately crossed with tomato mice (B6.Cg-Gt(ROSA)26Sortm14(CAG-tdTomato)HzeJ) (Bar Harbor, USA), where Cre excision results in tdTomato expression in *Pf4* expressing cells. Mice ubiquitously expressing nuclear green fluorescent protein (NZeg-eGFP) were also used. Mice of both sexes between the ages of 6–12 weeks were used. All experimental work was approved by the MARP Ethics Committee, Monash University, Australia and conducted in compliance with the specified ethics regulations.

### Human bone marrow

Fresh human marrow (BM) was collected from consenting patients undergoing hip replacement surgery at St Vincent’s Private Hospital Melbourne. All experimental work was approved by St Vincent’s Hospital Melbourne and St Vincent’s Private Hospital Melbourne Ethics committees and conducted in compliance with the specified ethics regulations. Informed consent was obtained from all participants. Cells were washed in PBS and filtered using a 100-μm strainer. Mature BM cells were depleted using anti-human CD11b, CD14, CD20 (see Supplementary Table 1) and Dynabeads (Thermo Fisher Scientific, USA) then stained with 10 μM Hoechst 33342 for 45 min at 37 °C followed by anti-human CD41-AF700 (or APC). Red blood cells (RBC) were lysed in 0.15 M ammonium chloride for 1 min before analysis/isolation on an Influx using a 100-μm nozzle at 20 psi. Bright-field and fluorescent images were acquired using an Olympus IX71 or BX51 microscope with a DP70 camera (Japan). DP controller 2.1.1.183 was used to collect bright-field and fluorescent microscopy images; MetaMorph version 7.7.8.0 or Fiji/ImageJ versions 1.52p and 1.52n were used for image analysis. Cytoplasmic and nuclear areas of LCM and SCM were measured with ImageJ or the region measurement tool of MetaMorph image analysis software (Molecular Devices, USA).

### Murine tissue processing

Murine PB was collected and RBC was lysed. Central BM (cBM), endosteal BM (eBM) or total BM was isolated as previously described^84^. Briefly, cBM was collected by removing the metaphyseal regions and flushing the tibias, femurs (with a 21 gauge) and pelvic bones with a (23 gauge) needle; eBM was collected by crushing flushed bones and metaphyseal regions before treatment for 5 min at 37 °C with Dispase neutral protease grade II from Bacillus Polymyxa (2.75 mg/ml; Roche Diagnostics, Basel, Switzerland) and Collagenase Type I from Clostridium histolyicum (2.1 mg/ml; Worthington Biochemical, Lakewood, USA); total BM was collected by crushing whole leg bones before enzymatic digestion as per eBM. Post-enzymatic treatment, cells were washed and passed through a 40 µm (for HSC) or 100 µm strainer (for MK). Spleens were disaggregated using a 40 µm cell strainer. Cell counts were performed using a Sysmex KX-21N (Sysmex, Japan) or CELL-DYN Emerald (Abbott, USA) automated cell counter.

### Megakaryocyte and platelet isolation and analysis

Murine MK were analysed and prospectively isolated as previously described^48^. Briefly, lineage-depleted cBM were stained with 10 μM Hoechst 33342 for 45 min at 37 °C then stained with anti-mouse CD41-AF700 (CD61-PE or c-MPL-biotin followed by SAV-AF647, see Supplementary Table 2). In this study, MK were always identified and/or isolated using the combination of antigen expression (CD41, CD61 or c-MPL) and DNA content (ploidy) to identify MK of high ploidy. Analysis was performed on an LSR II (BD Biosciences, USA) and MK were isolated using a 100-μm nozzle at 20 psi (Influx, BD Biosciences, USA, BD FACS Software 1.2.0.142 was used for FACS.). To collect platelet-rich plasma (PRP), whole blood was spun at 130 g for 8 min and supernatant washed in PBS. For platelet analysis, whole blood or PRP was stained with 5 μg/ml CD41-AF700. Anti-platelet serum (Cedarlane, CLA31440, Canada) was diluted 1/20 in PBS and injected into the peritoneum.

### Prolonged platelet depletion in MK-LCM^WT/WT^ mice

MK-LCM^WT/WT^ mice were injected with APS once every 3 days (days 1, 4 and 7) and HSC was assessed at day 9.

### Platelet infusion into MK-LCM^Δ/Δ^ mice to increase circulating platelets

1 × 10^9^ MK-LCM^WT/WT^ platelets were injected IV into MK-LCM^Δ/Δ^ mice on day 1 and 0.75 × 10^9^ platelets were infused each subsequent day for 5 more days before HSC was assessed at day 7.

### MK transplant and platelet formation assay

High ploidy RFP^+^ LCM or SCM were injected into the tail vein of C57Bl/6 mice with 2 × 10^5^ non-irradiated whole BM cells^85^. Donor platelets were identified as CD41^+^RFP^+^.

### Hematopoietic stem and progenitor isolation and analysis

Murine lineage-committed cells were analysed in cBM, eBM, spleen and PB using CD3, B220, Gr-1 and Mac-1, hematopoietic stem and progenitors were analysed as lineage negative (Lin^-^) Sca1^+^c-Kit^+^ cells, HSC were defined as Lin^-^Sca1^+^c-Kit^+^CD150^+^CD48^-^ cells (see Supplementary Table 2). BOP (*N-*(benzenesulfonyl)-_L_-prolyl-_L_-*O*-(1-pyrrolidinylcarbonyl)tyrosine), α_4_, α_9_ and CXCR-4 staining were performed as previously described^40^. Cells were treated with R-BC154 (100 nM) in PBS (0.5% BSA) containing no exogenous cations or with 1 mM CaCl_2_/MgCl_2_. For competitive displacement of in situ-bound R-BC154, WBM cells were treated with 1000 nM BOP in PBS containing 0.5% BSA ± 1 mM CaCl_2_/MgCl_2_ for 45 min at 37 °C before flow cytometric analysis. For the assessment of α_9_β_1_ contribution to R-BC154 binding, the selective α_4_β_1_ antagonist BIO5192 (1000 nM) or dual α_4_β_1_/α_9_β_1_ antagonist BOP (1000 nM) in PBS containing 0.5% BSA ± 1 mM CaCl_2_/MgCl_2_ at 37 °C for 30 min. Cells were washed, stained with cell surface markers for HSPC analysed by flow cytometry. The contribution of α_9_β_1_ binding was calculated by measuring the difference in MFI between R-BC154 plus BIO5192 and R-BC154 plus BOP and was expressed as a percentage of R-BC154 alone. Cell cycle analysis was performed using Ki67 and Hoechst 33342. All flow cytometric data were collected using an LSR II (BD Biosciences, USA, BD FACSDIVA Software 8.0.1 and analysed using FlowJo v10.) and hematopoietic stem and progenitors were sorted on an Influx with a 70μm nozzle (BD Biosciences, USA) following a density gradient (1.077 g/cm^3^) and lineage (CD3, B220, Gr-1, Mac-1 and Ter119) depletion using sheep anti-rat IgG Dynabeads as previously described^84^. A bead-to-cell ratio of 1:3 was used as per the manufacturer’s instructions.

### Transplant assays

In homing assays, 6–12k GFP^+^ MK-LCM^WT/WT^ or 14–16k RFP^+^ MK-LCM^Δ/Δ^ total HSC were transplanted intravenously with 2 × 10^5^ non-irradiated WT whole BM cells into non-irradiated C57BL/6 mice and BM assessed 15 h post-transplant. Homing efficiency was calculated as previously described^86^. The percentage of white blood cells was determined per sample and the denominator (*D*) was mathematically calculated as the total number of WBC cells assessed (*D* = % WBC / 100 × measured events). The proportion of donor (% do) of analysed BM was calculated using the number of positive events observed (% do = # positive events / *D* × 100). Per recipient, the total number of donor cells detected in the BM was calculated using the proportion of donor cells, the total number of BM cells isolated and the assumption that one femur, tibia, and pelvic bone represents 15% of the total number of cells in the mouse (total # do = (% do / 100 × #BM) / 15 × 100). Finally, the proportion of cells homed was calculated using the total number of donor cells and the number of transplanted (# cells injected) into each recipient (% homed = total # do / # cells injected × 100). In all transplant assays, cells were injected intravenously with 2 × 10^5^ irradiated WT whole BM cells (15 Gy) into lethally irradiated C57BL/6 recipients (10 Gy, split dose of 5 Gy, 4 h apart, 1 day prior to transplant). To assess the hematopoietic potential of HSC isolated from a microenvironment with diminished LCM, 5800 RFP^+^ MK-LCM^Δ/Δ^ HSC were transplanted with or without 5800 GFP^+^ MK-LCM^WT/WT^ HSC. In limiting dilution engraftment transplant assays, 10, 30, 100, 300, 1k, 3k or 10k RFP^+^ MK-LCM^Δ/Δ^ HSC or 10, 30, 100, 300 RFP^+^ MK-LCM^WT/WT^ HSC were transplanted into different recipient mice. In competitive limiting dilution engraftment transplant assays, 30 GFP^+^ MK-LCM^WT/WT^ HSC were competed against 10, 30, 100 or 300 RFP^+^ MK-LCM^Δ/Δ^ HSC. Mice were bled 6, 12 and 20 weeks after transplant for donor platelet and lineage analysis, and at 20 weeks after transplant, BM and spleen were also assessed for hematopoietic stem and progenitors and MK.

### Proliferation assay

Fifty endosteal HSC were co-cultured with 50 high ploidy LCM or SCM in SFEMII (StemCell Technologies) with L-glutamine (2 mM/ml, Life Technologies), human IL-11 (100 ng/ml, Chemicon, Australia), mouse stem cell factor (10 ng/ml, Chemicon), human IL-6 (10 ng/ml, Peprotech, USA), human FLT-3 ligand (10 ng/ml, Apollo Cytokine Research, Australia), mouse IL-3 (133 U/ml, Immunex Corporation, USA) and human thrombopoietin (TPO, 5 ng/ml, Apollo Cytokine Research) for 7 days. Manual cell counts were then performed.

### Transmission electron microscopy (TEM)

Isolated MK were processed as per Kumar et al.^87^. Briefly, 5 × 10^6^ RBC were pelleted with isolated MK and fixed in 3% PFA, 2.5% glutaraldehyde in 0.1 M sodium cacodylate buffer (SCB) before staining with Evans Blue to visualise the pellet. Cells were embedded in 4% agarose in 0.1 M SCB, removed from the tube and trimmed. Agarose-embedded cell pellets were post-fixed in 1% osmium tetroxide in 0.1 M SCB and dehydrated with increasing concentrations of ethanol followed by embedding in Spurr’s resin with sequential changes in 3:1, 1:1 and 1:3 ethanol:Spurr’s Resin, followed by immersion and embedding in pure resin and polymerising overnight at 60 °C. Ultrathin 70 nm sections were cut with a diamond knife, collected onto thin bar copper grids and stained with uranyl acetate (1 in 15 dilution in water) followed by Reynolds Lead Citrate. Grids were assessed using a Tecnai 12 transmission electron microscope (FEI, The Netherlands) operating at a voltage of 120 KV and images were acquired with an FEI Eagle 4kx4k CCD camera. AnalySIS v3.2 camera control software was used to acquire TEM images.

### Real-time PCR

Four hundred MK were lysed with water, RNA isolated and cDNA generated using Cells-to-Ct kit (Thermo Fisher), and assayed with *Rnc2* Taqman Assay (Mm04260181_s1, Thermo Fisher Scientific, USA), mouse *Tfrc* Gene Copy Reference Assay (Thermo Fisher Scientific Cat# 4458367) and TaqPath ProAmp Master Mix (Thermo Fisher Scientific Cat# A30865) in an Applied Biosystems 7500 Real-Time PCR System. ddCt was calculated using *Tfrc* as control, *Rnc2* is located within the unique mitochondrial DNA sequence^88^, relative copy numbers of mtDNA were calculated using ddCT and ploidy of the cells: Relative mtDNA Copy Number = 2^-ddCt^ × ploidy/2. TaqMan Assays (Thermo Fisher) 4352933E (*Actb*, housekeeper), Mm00494336_m1 (*Zfpm1/Fog-1*), Mm00801891_m1 (*Nfe2*), Mm01352636_m1 (*Gata1*), Mm00492301_m1 (*Gata2*) and Mm00437040_m1 (*Tpo*) were also used. Applied Biosystems 7500 System Software v1.5.1 was used for real-time PCR data collection.

### ELISA

Plasma was collected from whole blood centrifuged at 2000g for 20 min. cBM fluid was collected by flushing leg bones in 250 µl PBS. Flushed bones were then crushed in 200 µl PBS to collect eBM fluid. Lysate samples were generated by lysing sorted cells or crushing in lysis buffer (1% NP40, 1× Protease inhibitors (Roche, Switzerland), 1 mM AEBSF, 1 mM Na_3_VO_4_, 50 mM TrisHCl, 150 mM NaCl, 1 mM EDTA). MK conditioned media was generated from the culture of 2000 LCM or SCM in 100 µl IMDM, 1% BSA (Sigma) and 5 ng/ml hTPO for 3 days. ELISAs were performed as per the manufacturer’s instructions (PF4 (MCX400, R&D Systems, USA), TPO (MTP00, R&D Systems, USA), FX (MFXKT-TOT, Molecular Innovations, USA), TGFβ1 (MB100B, R&D Systems, USA), SCF (MCK00, R&D Systems, USA), MMP-9 (MMPT90, R&D Systems, USA), SDF-1 (MCX120), FGF-1 (Abcam, ab223587, USA) and tcOPN (27259, IBL, Japan) and data were recorded using an EnSpire 2300 Multilabel Reader, EnSpire Manager 4.13.3005.1482 (PerkinElmer) was used to assess ELISA results.

### Cytokine array

Bones from one hind limb were flushed into 250 µl of PBS, cells were pelleted and counted, and the supernatant was sent to CruxBiolabs (Australia) for analysis using a custom Quantibody Assay (RayBiotech). Data were normalised to cell number. GenePix4000B microarray scanner (Molecular Devices) for cytokine array data collection, GenePixPro version 4.0.0.54 was used for cytokine array data analysis.

### Mass spectrometry sample preparation and nUPLC-MS/MS analysis

Bones from two hind limbs were flushed in 1 ml PBS supplemented with cOmplete Protease Inhibitor Cocktail (Roche), 1 mM AEBSF and 1 mM Na_3_VO_4_ (Sigma). For quantitative proteomics experiments, samples were digested using a single-pot solid-phase-enhanced sample preparation method^89^. Protein samples (50 μg) were reduced with 5 mM TCEP (Sigma) for 15 min, alkylated for 30 min with 50 mM iodoacetamide (Sigma) and digested with 1 μg trypsin gold (Promega) for 16 h at 37 °C. Peptide preparations were resuspended in 100 μl 0.1% FA and 3 μl aliquots separated in triplicate using a two-column chromatography setup comprising a PepMap100 C18 20 mm × 75 μm trap and a PepMap C18 500 mm × 75 μm analytical column (Thermo Fisher). Samples were concentrated onto the trap column at 5 μl/min for 5 min and infused into an Orbitrap Fusion Lumos Tribrid mass spectrometer (Thermo Fisher) at 300 nl/min running 120 min gradients from 99% Buffer A (0.1% FA) to 40% Buffer B (99% acetonitrile, 0.1% FA) on a Dionex Ultimate 3000 UPLC system (Thermo Fisher). The Lumos mass spectrometer was operated in data-dependent mode, automatically switching between the acquisition of a single Orbitrap MS scan (resolution 120,000) every 3 s and the top-20 multiply charged precursors selected for EThcD fragmentation (maximum fill time, 100 m; AGC of 5 × 104 with a resolution of 30,000 for Orbitrap MS-MS scans).

### Mass spectra database searching and label-free quantification

Peptide identification and label-free quantification were accomplished using MaxQuant version 1.6.0.16^90^. Database searches were performed against a protein database containing Swiss-Prot and TrEMBL sequences for *Homo sapiens* and *Mus Musculus*, as well as common proteomics contaminants (123,757 entries). High-resolution MS/MS data were searched with trypsin cleavage specificity allowing for two miscleavage events, carbamidomethylation of cysteine (+57 Da) set as a fixed modification, and oxidation of methionine (+16 Da), acetylation of protein N termini (+42 Da), as well as pyroglutamylation of N-terminal glutamine (−17 Da) or glutamate (−18 Da) residues as variable modifications. The precursor mass tolerance was set to 20 ppm for the first search and 10 ppm for the main search with a maximum false discovery rate of 1.0% set for protein and peptide identifications. To enhance the identification of peptides between samples, the match between runs option was enabled with the precursor match window set to 2 min and an alignment window of 10 min in addition to the re-quantification module. Statistical analysis of label-free MS-based quantitative proteomics data was based on three biological replicates (3 × MK-LC^WT/WT^ vs 3 × MC-CL^Δ/Δ^) and three technical replicates (18 samples in total) using the peptide-level robust ridge regression method for relative protein quantification using the open-source R package MSqRob version 0.7.6^91,92^. The label-free quantitative proteomics data have been deposited into the ProteomeXchange Consortium (http://proteomecentral.proteomexchange.org) via the PRIDE partner repository with the dataset identifier PXD040423.

### Mitochondria

Cell-Tak (22.5 μg/ml, Corning, USA) was dropped onto FluoroDishes (World Precision Instruments, USA) and allowed to adhere for 20 min. Fresh MK, LSK or platelets were either (1) stained with MitoTrackerGreen (25 nM) for 10 min, washed and dropped onto Cell-Tak for imaging or (2) dropped onto Cell-Tak, fixed in 4% PFA, permeabilized with PBS/0.3% Triton X-100, washed in PBS/0.05% Tween 20 and incubated in anti-Tom20/PBST/3%BSA (1:400, rabbit polyclonal, Santa Cruz, SC-11415, USA, see Supplementary Table 2) for 90 min at room temperature. Washed samples were incubated with anti-rabbit-IgG AF568 (Molecular Probes, USA) for 1 h and assessed using confocal microscopy on a Leica TCS SP8 confocal microscope (Leica Microsystems, Germany) equipped with HyD detectors and using a 63×/1.40NA oil immersion objective (HC PLAPO, CS2, Leica Microsystems, Germany). Microscopy data were recorded using Leica LAS X Life software in 1024 × 1024 pixel format, 400 Hz scan speed, and 3× line accumulation mode. Z-sectioning was performed using 250 nm slices. Confocal images were collected using Leica LAS X Life Software version 3.5.5.19976. Leica.lif files were converted to multi-colour.tiff composite stacks using custom-written Fiji/ImageJ macros. Images were analysed using Fiji and custom-written macros^93^. Mitochondrial image stacks were filtered with a Gaussian Blur (2 pixels) and converted to binary image files using the Otsu Thresholding method. Segmented image objects were analysed using the ‘Analyze Particles’ function in Fiji/ImageJ (USA). The measurements of each image stack were saved as.csv files for further evaluation.

### Analysis of mitochondrial respiration

The mitochondrial activity of freshly isolated hematopoietic stem and progenitors was measured using a seahorse metabolic influx assay (AgilentTechnologies, USA, Agilent Seahorse Wave Desktop Version 2.2.1.5 was used). Fifteen thousand cells per well were resuspended in a serum-free seahorse XF DMEM medium (pH7.4 supplemented with 25 mM glucose and 1 mM pyruvate) and plated onto a 96-well plate pre-coated with Cell-Tak (22.4 μg/ml, Corning, USA). The plate was analysed immediately according to the manufacturer’s instructions using a seahorse Bioscience XFe96 extracellular flux analyser (AgilentTechnologies, USA). The oxygen consumption rate (OCR) was measured at 37 °C under basal conditions and in response to 1 μM oligomycin, 2 μM fluoro-carbonyl cyanide phenylhydrazone (FCCP) and 1 μM retone/antimycin A (AgilentTechnologies, USA). The seahorse assay was run in triplicate with the following assay conditions: 7 min mix time and 4 min read time. The OCR from cells were normalised against total protein measured by a DC protein assay (Bio-Rad, USA).

### Statistical analysis

Data were assessed for normal distribution with a Shapiro–Wilk normality test using GraphPad Prism 8 (GraphPad Software Inc, San Diego, USA) and presented as mean ± standard error of the mean. Differences between means were evaluated by an unpaired two-sided Student’s *t*-test or one-way ANOVA with a post hoc multiple comparisons test (Tukey’s multiple comparison test) if data were normally distributed and the variance was equal. Non-parametric Mann–Whitney or Kruskal–Wallis test (with Dunn’s multiple comparison analysis) was used if data were not normally distributed or if the unequal variance between groups was observed. Two-way ANOVA with Geisser–Greenhouse correction and post hoc multiple comparisons tests (Holm–Sidak multiple comparisons test) was also used where appropriate. The difference between groups is considered statistically significant if *p* < 0.05.

### Reporting summary

Further information on research design is available in the Nature Portfolio Reporting Summary linked to this article.

## Supplementary information

Supplementary Information

Reporting Summary

## Data Availability

The source data underlying Figs. 1a, b, e–h, j, k, m–o, 2b–c, e–s, 3a–e, 4a, b, d–k, m, n and 5e, g–i, n–r, t and Supplementary Figs. 1c–f, h–m, 2a–u,  3a–r and 4a, b are provided as a Source Data File. Additional information such as antibodies used in this study is provided in the Supplementary information. Additional data that support the findings of this study are available from the corresponding author upon reasonable request. Proteomics data have been deposited in the ProteomeXchange Consortium (http://proteomecentral.proteomexchange.org) via the PRIDE partner repository with the dataset identifier PXD040423. Source Data are provided with this paper.

## Source data

Source Data
