# Supplementary material for: High ploidy large cytoplasmic megakaryocytes are hematopoietic stem cells regulators and essential for platelet production
Source: Nat Commun. 2023 Apr 13;14:2099. doi: 10.1038/s41467-023-37780-7 (PMC10102126; doi:10.1038/s41467-023-37780-7)
Supplement: Supplementary file 1 — Supplementary Information [file 41467_2023_37780_MOESM1_ESM.pdf]

SUPPLEMENTARY INFORMATION

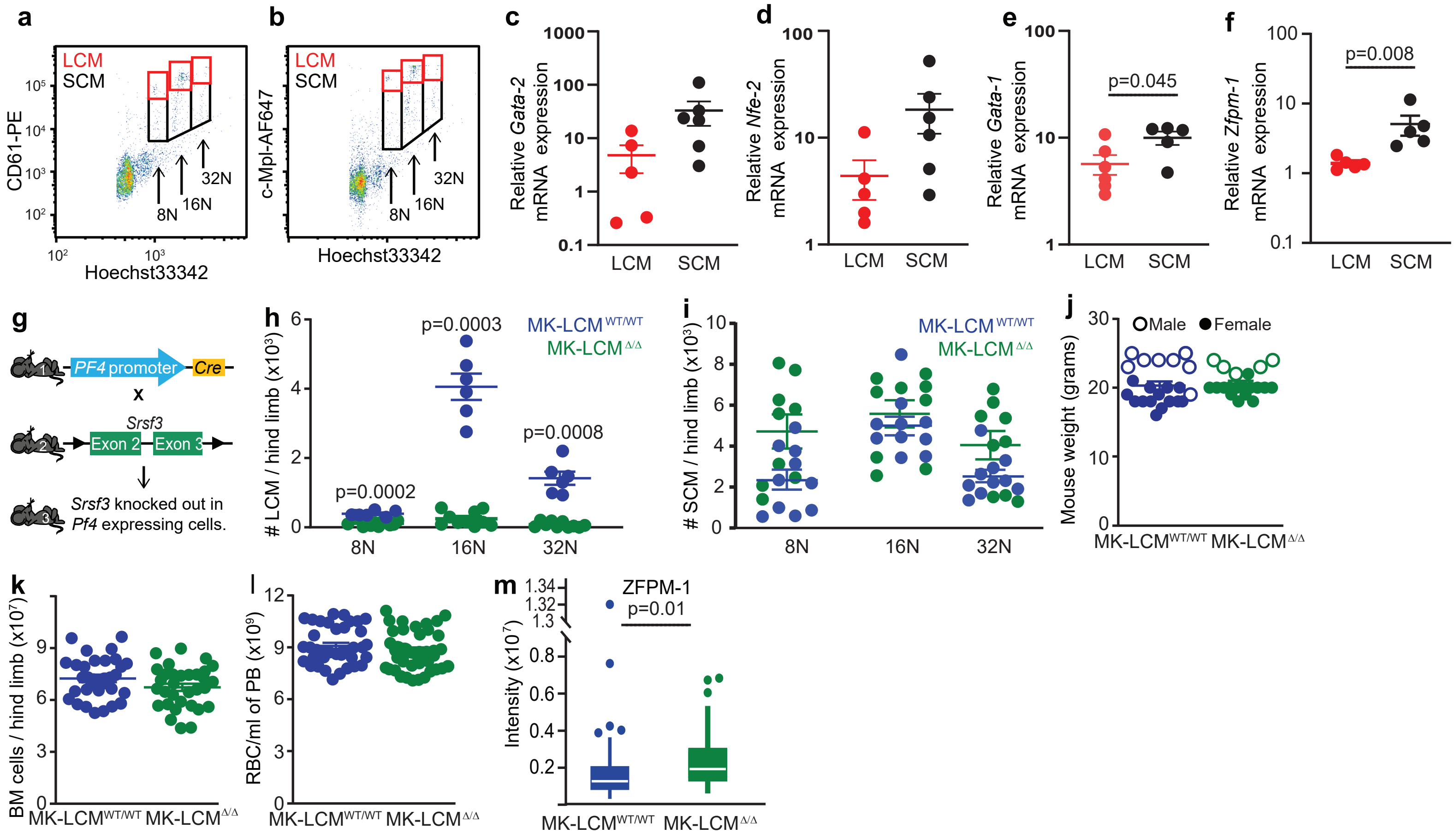

a
LCM
SCM
CD61-PE
Hoechst33342
8N
16N
32N
b
LCM
SCM
c-Mpl-AF647
Hoechst33342
8N
16N
32N
c
Relative Gata-2 mRNA expression
LCM
SCM
d
Relative Nfe-2 mRNA expression
LCM
SCM
e
Relative Gata-1 mRNA expression
p=0.045
LCM
SCM
f
Relative Zfpm-1 mRNA expression
p=0.008
LCM
SCM
g
PF4 promoter
Cre
x
Srsf3
Exon 2
Exon 3
Srsf3 knocked out in Pf4 expressing cells.
h
# LCM / hind limb (x10³)
p=0.0002
p=0.0003
p=0.0008
MK-LCM WT/WT
MK-LCM Δ/Δ
8N
16N
32N
i
SCM / hind limb (x10³)
MK-LCM WT/WT
MK-LCM Δ/Δ
8N
16N
32N
j
Male
Female
Mouse weight (grams)
MK-LCM WT/WT
MK-LCM Δ/Δ
k
BM cells / hind limb (x10⁷)
MK-LCM WT/WT
MK-LCM Δ/Δ
l
RBC/ml of PB (x10⁹)
MK-LCM WT/WT
MK-LCM Δ/Δ
m
ZFPM-1
p=0.01
Intensity (x10⁷)
MK-LCM WT/WT
MK-LCM Δ/Δ

Supplementary Fig. 1: MK of single ploidy exist as two distinct sub-populations.

(a) Representative flow cytometric plot of MK separated by ploidy and further sub-fractionated using CD61 into CD61^{bright} (LCM) and CD61^{dim} (SCM). 8N LCM = 0.15%, 8N SCM = 0.38%, 16N LCM = 0.68%, 16N SCM = 0.84%, 32N LCM = 0.07%, 32N SCM = 0.35%. **(b)** c-MPL can also be used to separate MK into LCM and SCM. 8N LCM = 0.12%, 8N SCM = 0.44%, 16N LCM = 0.8%, 16N SCM = 0.91%, 32N LCM = 0.07%, 32N SCM = 0.4%. (**a,b:** representative plots from at least 7 biological replicates from ≥2 experiments). Percentages shown in flow plots are current gate as a percentage of the parent. **(c)** RT-PCR analysis of *Gata-2* expression in 16N LCM and 16N SCM (MK-LCM$^{WT/WT}$ n = 5, MK-LCM$^{\Delta/\Delta}$ n = 6, biological replicates). **(d)** RT-PCR analysis of *Nfe-2* expression in 16N LCM and 16N SCM (MK-LCM$^{WT/WT}$ n = 5, MK-LCM$^{\Delta/\Delta}$ n = 6, biological replicates). **(e)** RT-PCR analysis of *Gata-1* expression in 16N LCM and 16N SCM (MK-LCM$^{WT/WT}$ n = 6, MK-LCM$^{\Delta/\Delta}$ n = 5, biological replicates). **(f)** RT-PCR analysis of *Zfpm-1* expression in 16N LCM and 16N SCM (MK-LCM$^{WT/WT}$ n = 5, MK-LCM$^{\Delta/\Delta}$ n = 5, biological replicates). (**c-f:** Data relative to HSC mRNA expression of each target gene, from 2 experiments per gene). **(g)** MK-LCM$^{\Delta/\Delta}$, *Pf-4-Cre-Srsf3* KO mouse model. **(h)** Absolute number of LCM per mouse hind limb (MK-LCM$^{WT/WT}$ n = 6, MK-LCM$^{\Delta/\Delta}$ n = 10, biological replicates from ≥2 experiments). **(i)** Absolute number of SCM per hind limb (MK-LCM$^{WT/WT}$ n = 10, MK-LCM$^{\Delta/\Delta}$ n = 9, biological replicates from ≥2 experiments). **(j)** Body weights of MK-LCM$^{WT/WT}$ and MK-LCM$^{\Delta/\Delta}$ mice (Female: MK-LCM$^{WT/WT}$ n = 14, MK-LCM$^{\Delta/\Delta}$ n = 15, Male: MK-LCM$^{WT/WT}$ n = 8, MK-LCM$^{\Delta/\Delta}$ n = 5, biological replicates from 8 experiments). (**k**) BM cellularity in the hind limb of MK-LCM$^{WT/WT}$ and MK-LCM$^{\Delta/\Delta}$ mice (MK-LCM$^{WT/WT}$ n = 31, MK-LCM$^{\Delta/\Delta}$ n = 30, biological replicates from 8 experiments). **(l)** Red blood cell counts in MK-LCM$^{WT/WT}$ and MK-LCM$^{\Delta/\Delta}$ mice (MK-LCM$^{WT/WT}$ n = 38, MK-LCM$^{\Delta/\Delta}$ n = 39, biological replicates from 8 experiments). **(m)** ZFPM-1 protein level in MK-

$LCM^{WT/WT}$ and MK-$LCM^{\Delta/\Delta}$ BM. Data generated in MSqRob (n =3 biological replicates from 1 experiment, assessed 9 different peptide sequences, $\geq$ 45 reads per sample group, unpaired two-sided Student's t-test). Mean (line), $1^{st}/3^{rd}$ quartiles (box), range (vertical line). Statistical analysis was performed using unpaired two-sided Student's t-test, p-values indicated for **(e),** unpaired two-sided Mann-Whitney test **(f),** Two-Way ANOVA with Geisser-Greenhouse correction, p<0.0001 (overall) with individual groups for each timepoint compared using Holm-Sidak multiple comparisons test, p-values indicated for **(h)**. Source data are provided as a Source Data File. Error bars = SEM.

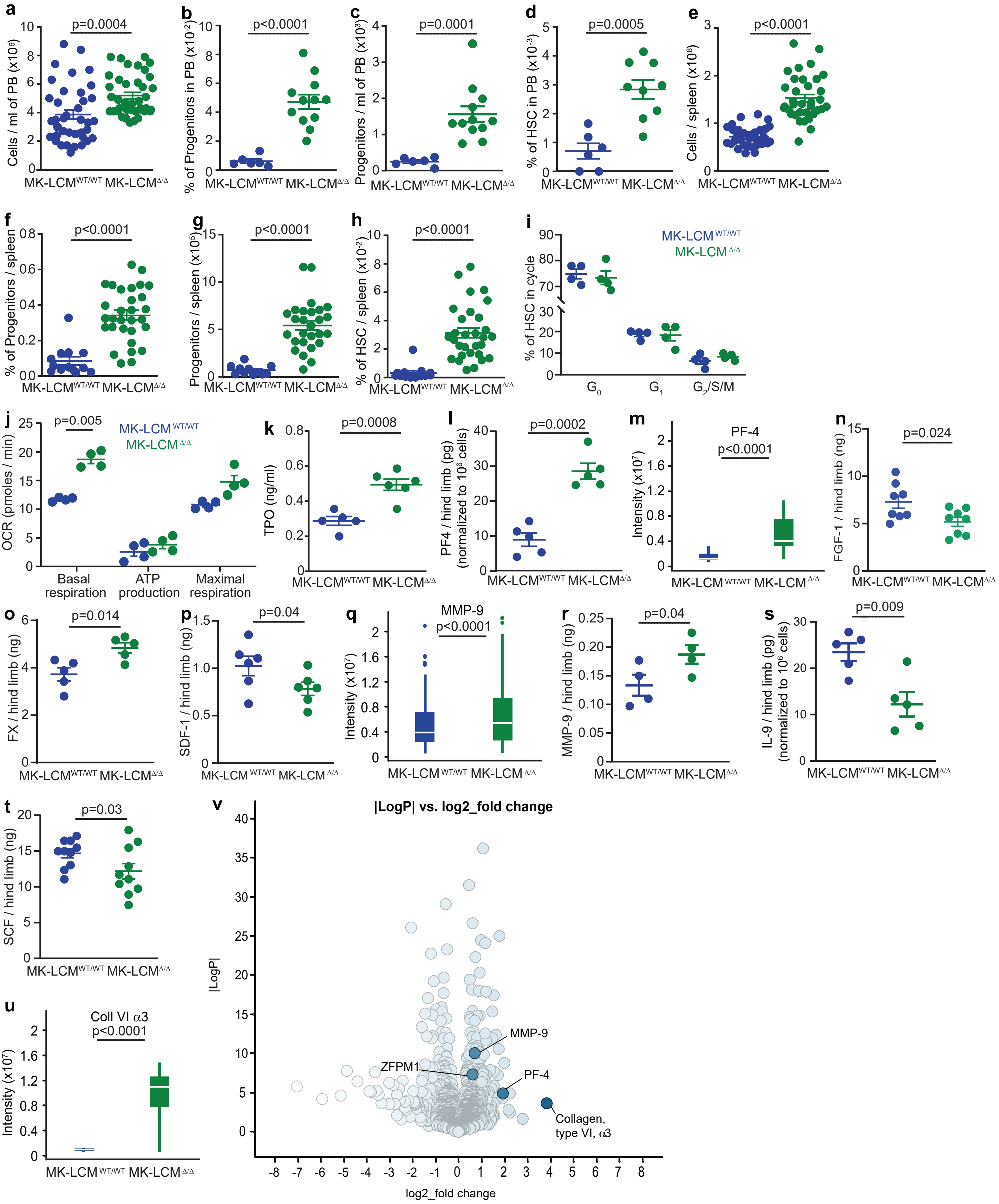

a
p=0.0004
Cells / ml of PB (x10^6)
MK-LCM^WT/WT MK-LCM^Δ/Δ
b
p<0.0001
% of Progenitors in PB (x10^-2)
c
p=0.0001
Progenitors / ml of PB (x10^3)
d
p=0.0005
% of HSC in PB (x10^-3)
e
p<0.0001
Cells / spleen (x10^8)
f
p<0.0001
% of Progenitors / spleen
g
p<0.0001
Progenitors / spleen (x10^5)
h
p<0.0001
% of HSC / spleen (x10^-2)
i
MK-LCM^WT/WT
MK-LCM^Δ/Δ
% of HSC in cycle
G0
G1
G2/S/M
j
p=0.005
OCR (pmoles / min)
Basal respiration
ATP production
Maximal respiration
k
p=0.0008
TPO (ng/ml)
l
p=0.0002
PF4 / hind limb (pg) (normalized to 10^6 cells)
m
PF-4
p<0.0001
Intensity (x10^7)
n
p=0.024
FGF-1 / hind limb (ng)
o
p=0.014
FX / hind limb (ng)
p
p=0.04
SDF-1 / hind limb (ng)
q
MMP-9
p<0.0001
Intensity (x10^7)
r
p=0.04
MMP-9 / hind limb (ng)
s
p=0.009
IL-9 / hind limb (pg) (normalized to 10^6 cells)
t
p=0.03
SCF / hind limb (ng)
u
Coll VI α3
p<0.0001
Intensity (x10^7)
v
|LogP| vs. log2_fold change
|LogP|
MMP-9
ZFPM1
PF-4
Collagen, type VI, α3
log2_fold change

Supplementary Fig. 2: Murine model with diminished LCM in the BM.

(a) PB white cell count in MK-LCM$^{WT/WT}$ and MK-LCM$^{\Delta/\Delta}$ mice (MK-LCM$^{WT/WT}$ n = 38, MK-LCM$^{\Delta/\Delta}$ n = 39, biological replicates from 8 experiments). **(b)** Frequency of LSK in blood, **(c)** number of LSK in PB and **(d)** frequency of HSC in PB (**b,c:** MK-LCM$^{WT/WT}$ n = 6, MK-LCM$^{\Delta/\Delta}$ n = 12, **d:** MK-LCM$^{WT/WT}$ n = 6, MK-LCM$^{\Delta/\Delta}$ n = 9, biological replicates from ≥2 experiments). **(e)** Spleen cellularity in MK-LCM$^{WT/WT}$ and MK-LCM$^{\Delta/\Delta}$ mice (MK-LCM$^{WT/WT}$ n = 31, MK-LCM$^{\Delta/\Delta}$ n = 35, biological replicates from 7 experiments). **(f)** Frequency of LSK in spleen (MK-LCM$^{WT/WT}$ n = 13, MK-LCM$^{\Delta/\Delta}$ n = 30, biological replicates from 7 experiments), **(g)** number of LSK in spleen (MK-LCM$^{WT/WT}$ n = 11, MK-LCM$^{\Delta/\Delta}$ n = 27, biological replicates from 7 experiments) and **(h)** frequency of HSC in spleen (MK-LCM$^{WT/WT}$ n = 13, MK-LCM$^{\Delta/\Delta}$ n = 30, biological replicates from 7 experiments). **(i)** Frequency of cHSC cycling in MK-LCM$^{WT/WT}$ and MK-LCM$^{\Delta/\Delta}$ BM. **(j)** Metabolic activity of MK-LCM$^{WT/WT}$ and MK-LCM$^{\Delta/\Delta}$ hematopoietic stem and progenitors (seahorse assay). (**i,j:** n = 4, representative data of 2 experiments and eHSC data). **(k)** TPO concentration in the plasma of MK-LCM$^{WT/WT}$ and MK-LCM$^{\Delta/\Delta}$ mice (MK-LCM$^{WT/WT}$ n = 5, MK-LCM$^{\Delta/\Delta}$ n = 6, biological replicates from 2 experiments). **(l)** Cytokine array data showing PF4 levels in MK-LCM$^{WT/WT}$ and MK-LCM$^{\Delta/\Delta}$ BM fluid (MK-LCM$^{WT/WT}$ n = 5, MK-LCM$^{\Delta/\Delta}$ n = 5, biological replicates from 1 experiment). **(m)** Proteomic analysis for PF-4 in the BM of MK-LCM$^{WT/WT}$ and MK-LCM$^{\Delta/\Delta}$ mice. Data generated in MSqRob (n = 3 biological replicates from 1 experiment, assessed 2 different peptide sequences, ≥ 10 reads per sample group, unpaired two-sided Student's t-test). Mean (line), 1st/3rd quartiles (box), range (vertical line). **(n)** Concentration of FGF-1 in the BM fluid of MK-LCM$^{WT/WT}$ and MK-LCM$^{\Delta/\Delta}$ mice (LCM$^{WT/WT}$ n = 8, MK-LCM$^{\Delta/\Delta}$ n = 8, biological replicates from 2 experiments). **(o)** FX concentration in the BM of MK-LCM$^{WT/WT}$ and MK-LCM$^{\Delta/\Delta}$ mice (LCM$^{WT/WT}$ n = 5, MK-LCM$^{\Delta/\Delta}$ n = 5, biological replicates from 2 experiments). **(p)** Concentration of SDF-1 in the

BM fluid of MK-LCM$^{WT/WT}$ and MK-LCM$^{\Delta/\Delta}$ mice (LCM$^{WT/WT}$ n = 6, MK-LCM$^{\Delta/\Delta}$ n = 6, biological replicates from 2 experiments, one-tailed t-test). **(q)** MMP-9 protein in MK-LCM$^{WT/WT}$ and MK-LCM$^{\Delta/\Delta}$ BM. Proteomics data generated in MSqRob (n = 3 biological replicates from 1 experiment, assessed 13 different peptide sequences, $\geq$ 74 reads per sample group, unpaired two-sided Student's t-test). Mean (line), 1st/3rd quartiles (box), range (vertical line). **(r)** Concentration of MMP-9 in the BM fluid of MK-LCM$^{WT/WT}$ and MK-LCM$^{\Delta/\Delta}$ mice (ELISA, LCM$^{WT/WT}$ n = 4, MK-LCM$^{\Delta/\Delta}$ n = 4, biological replicates from 1 experiment, one-tailed t-test). **(s)** Cytokine array data analysing the concentration of IL-9 in the BM fluid of MK-LCM$^{WT/WT}$ and MK-LCM$^{\Delta/\Delta}$ mice (LCM$^{WT/WT}$ n = 5, MK-LCM$^{\Delta/\Delta}$ n = 5, biological replicates from 1 experiment). **(t)** Concentration of SCF in the BM fluid of MK-LCM$^{WT/WT}$ and MK-LCM$^{\Delta/\Delta}$ mice measured by ELISA (LCM$^{WT/WT}$ n = 10, MK-LCM$^{\Delta/\Delta}$ n = 10, biological replicates from 2 experiments, one-tailed t-test). **(u)** BM proteomics data for Coll VI α3, generated in MSqRob (n≥1 individual mouse from 1 experiment, assessed 2 different peptide sequences, ≥1 reads per sample group, unpaired two-sided Student's t-test). Mean (thin line), 1st/ 3rd quartiles (box), range (vertical line). **(v)** Volcano plot of protein changes in the BM fluid of MK-LCM$^{WT/WT}$ and MK-LCM$^{\Delta/\Delta}$ mice from 1 experiment. Statistical analysis was performed using unpaired two-sided Mann-Whitney test **(a,c,f,h),** unpaired two-sided Student's t-test, p-values indicated for **(b,d,e,g,k,l,n,o,s),** Two-Way ANOVA with Geisser-Greenhouse correction, p = 0.003 (overall) with individual groups for each timepoint compared using Holm-Sidak multiple comparisons test, p-values indicated for **(j)** or unpaired one-way t-test **(p,r,t)**. Source data are provided as a Source Data File. Error bars = SEM.

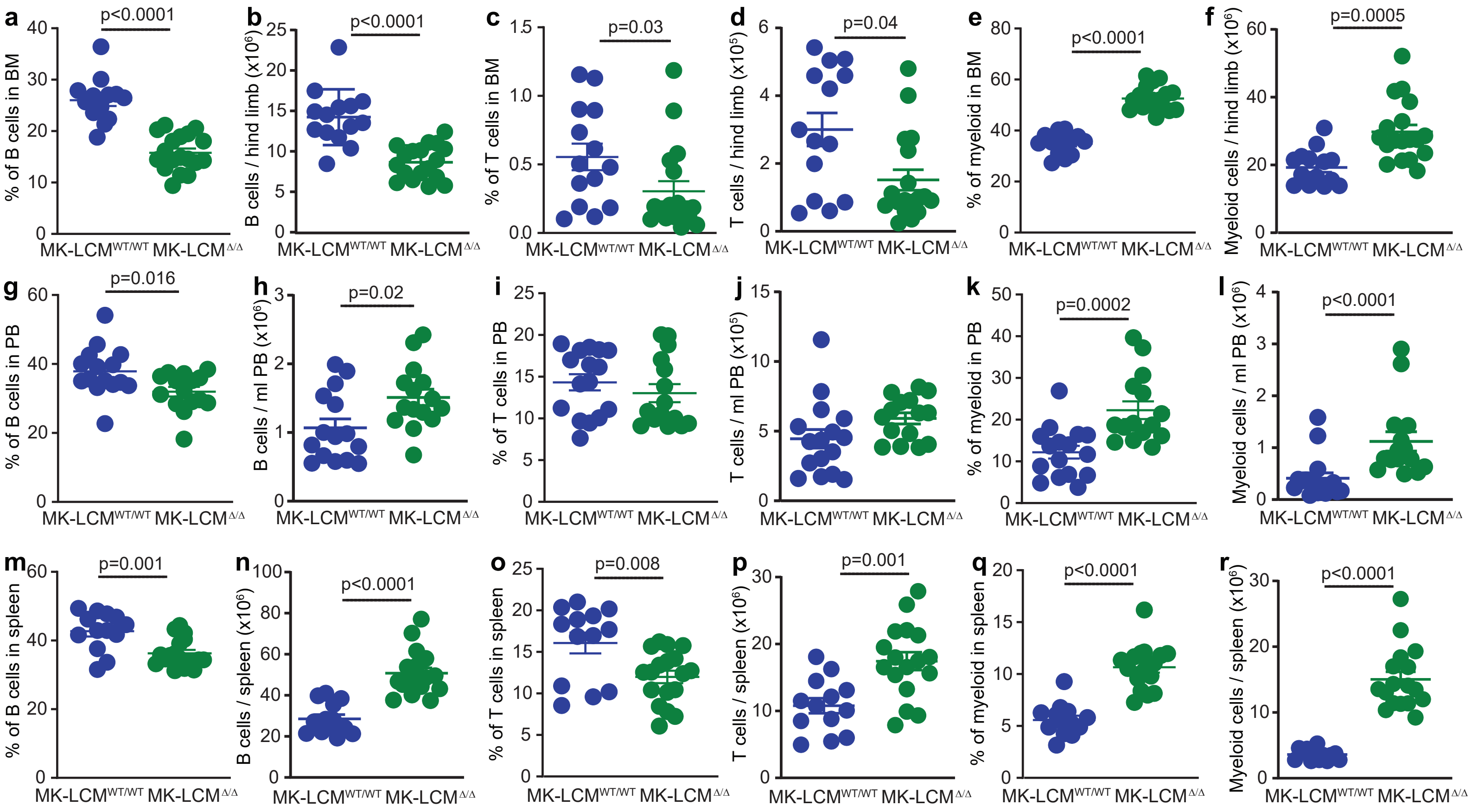

a
% of B cells in BM
p<0.0001
b
B cells / hind limb (x10^6)
p<0.0001
c
% of T cells in BM
p=0.03
d
T cells / hind limb (x10^5)
p=0.04
e
% of myeloid in BM
p<0.0001
f
Myeloid cells / hind limb (x10^6)
p=0.0005
g
% of B cells in PB
p=0.016
h
B cells / ml PB (x10^6)
p=0.02
i
% of T cells in PB
j
T cells / ml PB (x10^5)
k
% of myeloid in PB
p=0.0002
l
Myeloid cells / ml PB (x10^6)
p<0.0001
m
% of B cells in spleen
p=0.001
n
B cells / spleen (x10^6)
p<0.0001
o
% of T cells in spleen
p=0.008
p
T cells / spleen (x10^6)
p=0.001
q
% of myeloid in spleen
p<0.0001
r
Myeloid cells / spleen (x10^6)
p<0.0001
MK-LCMWT/WT
MK-LCMΔ/Δ

Supplementary Fig. 3: Loss of LCM results in decreased lymphoid and increased myeloid cell frequencies in the BM, PB and spleen.

(a-f) Frequencies and absolute numbers of B-, T- and myeloid cells in BM (LCM$^{WT/WT}$ n = 14, MK-LCM$^{\Delta/\Delta}$ n = 18, biological replicates from 4 experiments). **(g-l)** Frequencies and absolute numbers of B-, T- and myeloid cells in PB (LCM$^{WT/WT}$ n = 15, MK-LCM$^{\Delta/\Delta}$ n = 15, biological replicates, from 4 experiments). **(m-r)** Frequencies and absolute numbers of B-, T- and myeloid cells in spleen (LCM$^{WT/WT}$ n = 13, MK-LCM$^{\Delta/\Delta}$ n = 17, biological replicates, from 4 experiments). Statistical analysis was performed using unpaired two-sided Student's t-test, p-values indicated for **(a,b,e-g,m,n,p-r)** or unpaired two-sided Mann-Whitney test **(c,d,h,k,l,o).** Source data are provided as a Source Data File. Error bars = SEM.

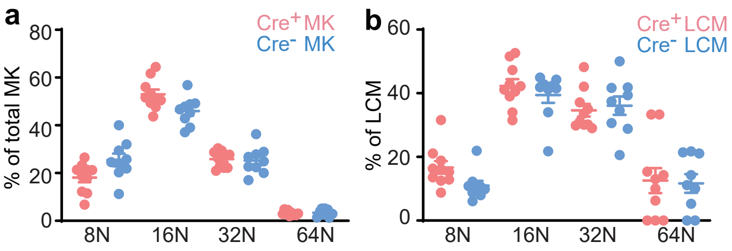
a
% of total MK
80
60
40
20
0
8N
16N
32N
64N
Cre+ MK
Cre- MK
b
% of LCM
60
40
20
0
8N
16N
32N
64N
Cre+ LCM
Cre- LCM

Supplementary Fig. 4: LCM and SCM populations

MK ploidy distribution **(a)** and frequency of LCM **(b)** in *Pf4-Cre Stop$^{fl/fl}$tdTomato* mice; where *Pf4* drives the expression of tdTomato in Cre^{+} mice (Cre^{+} n = 10, Cre^{-} n = 9, biological replicates, from 2 experiments). Statistical analysis was performed using Two-Way ANOVA (NS). Source data are provided as a Source Data File. Error bars = SEM.

Supplementary Table 1: Anti-human antibodies

Target Protein	Fluorophore	Clone	Isotype	Supplier	Cat #	Conc.
CD41	AF700<br><br>APC	HIP8<br><br>HIP8	Mouse IgG1<br>Mouse IgG1	Biolegend<br><br>BD	303728<br><br>559777	0.5µg/ml<br><br>0.3µg/ml
Mac-1, CD11b	Pure	ICRF44	Mouse IgG	BD	555386	1µg/ml
CD14	Pure	M5E2	Mouse IgG	BD	555396	1µg/ml
CD20	Pure	2H7	Mouse IgG2b	BD	555621	1µg/ml

Supplementary Table 2: Anti-mouse antibodies

Target Protein	Fluorophore	Clone	Isotype	Supplier	Cat #	Conc.
CD3e	BV510<br>APC Cy7	17A2<br>17A2	rat IgG2b<br>rat IgG2b	Biolegend<br>BD	100234<br>560590	2μg/ml<br>0.5μg/ml
CD45R, B220	APCCy7<br>BV510<br>AF647<br>purified	RA3_6B2<br>RA3_6B2<br>RA3_6B2<br>RA3_6B2	rat IgG2a<br>rat IgG2a<br>rat IgG2a<br>rat IgG2a	BD<br>BD<br>Biolegend<br>BD	552094<br>563103<br>103226<br>557390	0.5μg/ml<br>2μg/ml<br>1μg/ml<br>1μg/ml
Gr-1, Ly-6G/Ly-6C	APCCy7<br>AF647<br>purified	RB6-8C5<br>RB6-8C5<br>RB6-8C5	rat IgG2b<br>rat IgG2b<br>rat IgG2b	BD<br>Biolegend<br>BD	557661<br>108418<br>553123	0.1μg/ml<br>0.1μg/ml<br>1μg/ml
Mac-1, CD11b	APCCy7<br>AF647<br>purified	M1/70<br>M1/70<br>M1/70	rat IgG2b<br>rat IgG2b<br>rat IgG2b	BD<br>Biolegend<br>BD	557657<br>101218<br>553308	0.5μg/ml<br>0.1μg/ml<br>1μg/ml
CD117, c-Kit	BUV395<br>AF647	2B8<br>2B8	rat IgG2b<br>rat IgG2b	BD<br>Biolegend	564011<br>105818	1μg/ml<br>0.25μg/ml
Sca-1, Ly-6A/E	PECy7	E13-161.7	rat IgG2a	Biolegend	122514	0.4μg/ml
TER119	purified	TER119	rat IgG2b	BD	550565	0.1μg/ml
CD48	APC<br>FITC<br>BV421<br>BV510	HM48-1<br>HM48-1<br>HM48-1<br>HM48-1	A. Hamster IgG1	BD<br>BD<br>BD<br>BD	562746<br>557484<br>562745<br>563536	0.5μg/ml<br>1μg/ml<br>1μg/ml<br>1μg/ml
CD150, SLAMF1	BV650<br>PE<br>Biotin	TC15-12F12.2<br>TC15-12F12.2<br>TC15-12F12.2	rat IgG2a<br>rat IgG2a<br>rat IgG2a	Biolegend<br>Biolegend<br>Biolegend	115931<br>115904<br>115908	2μg/ml<br>1μg/ml<br>1μg/ml
CD41	AF700	MWReg30	Rat IgG1	Biolegend	133926	0.25μg/ml (MK)<br>5μg/ml (platelets)<br>10μg/ml (HSC)
CD61	PE	2C9.G2	A. Hamster IgG1	BD	553347	5μg/ml
c-Mpl	Biotin		Goat IgG	R&D	AF1317	2μg/ml
α_4	BV605	9C10	Rat IgG2a	BD	745183	2μg/ml
α_9	PE		Goat IgG	R&D	FAB3827P	0.5μg/ml
CXCR-4	AF647	L276F12	Rat gG2b	Biolegend	146503	2.5μg/ml

Tom20	Pure	FL-145	Rabbit IgG	Santa Cruz	SC-11415	0.5µg/ml
VWF	Pure		Rabbit IgG	Abcam	Ab9378	8µg/ml
Ki67	BV786	B56	Mouse IgG	BD	563756	1 in 10
Rabbit-IgG	AF568			Invitrogen	A-11011	10µg/ml
Goat-IgG	AF647			Molecular Probes	A21447	2µg/ml
SAV	AF647			Biolegend	405237	0.5µg/ml
SAV	BUV805			BD	564923	1µg/ml

Supplementary Table 3: Peptides used in the proteomics analysis:

MMP-9	DMIDDAFAR
	GSPLQGPFLTAR
	GVVIPTYYGNSNGAPCHFPFTFEGR
	LGLGPEVTHVSGLLPR
	QLSLPQTGELDSQTLK
	QPTFVVFPK
	QSLRPALLMLQK
	SLDKLGLGPEVTHVSGLLPR
	SQKVDPQSVIR
	TWPALPATLDSAFEDPQTKR
	VDKEFSGVPWNSHDIFQYQDK
	VDPQSVIR
	VFFFSGR
PF-4	HITSLEVIK
	TISSGIHLK
ZFPM-1	APAGAAAEPDPSR
	GEIYSPGAGHPAAK
	GPPAPAPAPGGGGGHR
	LQQGAGSSGAAGTPTGLFSGTK
	LVTEPHGAPR
	QAHGLQVAKPAASPGAEPR
	RPPAPTTAPGPAAPALTAPPVR
	VEAAEEPEATR
	VRGDLVEHLR
Col6a3	EVQVSEVTENSAR
	PAPAQPVLAK
