# Supplementary material for: High ploidy large cytoplasmic megakaryocytes are hematopoietic stem cells regulators and essential for platelet production
Source: Nat Commun. 2023 Apr 13;14:2099. doi: 10.1038/s41467-023-37780-7 (PMC10102126; doi:10.1038/s41467-023-37780-7)
Supplement: Supplementary file 2 — Reporting Summary [file 41467_2023_37780_MOESM2_ESM.pdf]

nature portfolio

Corresponding author(s): Susan Nilsson

Last updated by author(s): Feb 26, 2023

# Reporting Summary

Nature Portfolio wishes to improve the reproducibility of the work that we publish. This form provides structure for consistency and transparency in reporting. For further information on Nature Portfolio policies, see our Editorial Policies and the Editorial Policy Checklist.

## Statistics

For all statistical analyses, confirm that the following items are present in the figure legend, table legend, main text, or Methods section.

| n/a | Confirmed | |
|---|---|---|
| ☐ | ☒ | The exact sample size (*n*) for each experimental group/condition, given as a discrete number and unit of measurement |
| ☐ | ☒ | A statement on whether measurements were taken from distinct samples or whether the same sample was measured repeatedly |
| ☐ | ☒ | The statistical test(s) used AND whether they are one- or two-sided<br>*Only common tests should be described solely by name; describe more complex techniques in the Methods section.* |
| ☐ | ☒ | A description of all covariates tested |
| ☐ | ☒ | A description of any assumptions or corrections, such as tests of normality and adjustment for multiple comparisons |
| ☐ | ☒ | A full description of the statistical parameters including central tendency (e.g. means) or other basic estimates (e.g. regression coefficient) AND variation (e.g. standard deviation) or associated estimates of uncertainty (e.g. confidence intervals) |
| ☐ | ☒ | For null hypothesis testing, the test statistic (e.g. *F*, *t*, *r*) with confidence intervals, effect sizes, degrees of freedom and *P* value noted<br>*Give P values as exact values whenever suitable.* |
| ☒ | ☐ | For Bayesian analysis, information on the choice of priors and Markov chain Monte Carlo settings |
| ☒ | ☐ | For hierarchical and complex designs, identification of the appropriate level for tests and full reporting of outcomes |
| ☒ | ☐ | Estimates of effect sizes (e.g. Cohen's *d*, Pearson's *r*), indicating how they were calculated |

*Our web collection on statistics for biologists contains articles on many of the points above.*

## Software and code

Policy information about availability of computer code

| | |
|---|---|
| Data collection | BD FACS Sortware 1.2.0.142 was used for fluorescence activated cell sorting.<br>BD FACSDIVA Software 8.0.1 was used for flow cytometric analysis.<br>EnSpire Manager 4.13.3005.1482 (PerkinElmer) was used to assess ELISA results.<br>Agilent Seahorse Wave Desktop Version 2.2.1.5 was used for the seahorse assay.<br>Applied Biosystems 7500 System Software v1.5.1 was used for real-time-PCR data collection.<br>AnalySIS v3.2 camera control software was used to acquire TEM images.<br>Confocal images were collected using Leica LAS X Life Software version 3.5.5.19976.<br>DP controller 2.1.1.183 as used to collect bright field and fluorescent micrscopy images.<br>GenePix4000B microarray scanner (Molecular Devices) for cytokine array data collection.<br>Orbitrap Fusion Lumos Tribrid mass spectrometer (ThermoFisher) was used for proteomics data collection. |
| Data analysis | GraphPad Prism 8 (GraphPad software Inc, San Diego, USA) was used for statistical analysis.<br>FlowJo v10 was used for flow cytometric analysis.<br>Fiji/ImageJ versions 1.52p and 1.52n were used for image analysis.<br>MetaMorph version 7.7.8.0 was used for image analysis.<br>GenePixPro version 4.0.0.54 for cytokine array data analysis.<br>MaxQuant version 1.6.0.16 and MSqRob version 0.7.6 were used for quantitative proteomics data analysis. |

For manuscripts utilizing custom algorithms or software that are central to the research but not yet described in published literature, software must be made available to editors and reviewers. We strongly encourage code deposition in a community repository (e.g. GitHub). See the Nature Portfolio guidelines for submitting code & software for further information.

## Data

Policy information about availability of data

All manuscripts must include a data availability statement. This statement should provide the following information, where applicable:

- Accession codes, unique identifiers, or web links for publicly available datasets
- A description of any restrictions on data availability
- For clinical datasets or third party data, please ensure that the statement adheres to our policy

The datasets generated during and/or analysed during the current study are available in the manuscript, supplementary files, source data file, PROTEOMICS DATA REPOSITORY and from the corresponding author upon reasonable request.

## Human research participants

Policy information about studies involving human research participants and Sex and Gender in Research.

| | |
|---|---|
| Reporting on sex and gender | Bone marrow from consenting adults over the age of 18 years, irrespective of sex or ethnicity, undergoing hip replacement were used in this study. Due to confidentiality reasons, only sex and age information were given. Both male and female donor samples are presented in this article; donors were aged ~40-76 years old. This study assumed there was no sex-bias in MK sub-populations and in platelets formation. |
| Population characteristics | See above. |
| Recruitment | Appropriate samples (see above) will be identified by the Michelle Dowsey or Peter Choong at St Vincent's Hospital or St Vincent's Private Hospital. Generally it is the older population who require hip replacements. |
| Ethics oversight | All experimental work was approved by St Vincent's Hospital Melbourne and St Vincent's Private Hospital Melbourne Ethics committees and conducted in compliance with the specified ethics regulations. |

Note that full information on the approval of the study protocol must also be provided in the manuscript.

# Field-specific reporting

Please select the one below that is the best fit for your research. If you are not sure, read the appropriate sections before making your selection.

☒ Life sciences ☐ Behavioural & social sciences ☐ Ecological, evolutionary & environmental sciences

For a reference copy of the document with all sections, see nature.com/documents/nr-reporting-summary-flat.pdf

# Life sciences study design

All studies must disclose on these points even when the disclosure is negative.

| | |
|---|---|
| Sample size | Sample sizes were chosen to provide sufficient statistical power to detect differences. |
| Data exclusions | No data was excluded. |
| Replication | Data was replicated using multiple n, as well as biological repeats as stated in the manuscript. There were no situations of unreproducibility. |
| Randomization | All samples, including mice, were always randomized in experiments, and randomized into treatment groups where appropriate. |
| Blinding | Investigators performing the initial analysis and data collection were blinded as to which treatment group or strain a sample belonged to. |

# Reporting for specific materials, systems and methods

We require information from authors about some types of materials, experimental systems and methods used in many studies. Here, indicate whether each material, system or method listed is relevant to your study. If you are not sure if a list item applies to your research, read the appropriate section before selecting a response.

## Materials & experimental systems

| n/a | Involved in the study | |
|---|---|---|
| ☐ | ☒ | Antibodies |
| ☒ | ☐ | Eukaryotic cell lines |
| ☒ | ☐ | Palaeontology and archaeology |
| ☐ | ☒ | Animals and other organisms |
| ☒ | ☐ | Clinical data |
| ☒ | ☐ | Dual use research of concern |

## Methods

| n/a | Involved in the study | |
|---|---|---|
| ☒ | ☐ | ChIP-seq |
| ☐ | ☒ | Flow cytometry |
| ☒ | ☐ | MRI-based neuroimaging |

# Antibodies

| | |
|---|---|
| Antibodies used | The Supplier, catalogue number, clone, fluorophore and concentration of all antibodies used in the study are described in Supplementary Tables 1 and 2. |
| Validation | All antibodies were validated (against their matching isotype control) before use. |

# Animals and other research organisms

Policy information about studies involving animals; ARRIVE guidelines recommended for reporting animal research, and Sex and Gender in Research

| | |
|---|---|
| Laboratory animals | C57Bl/6 (MK-LCMWT/WT), mice carrying the floxed serine-arginine rich splicing factor 3 (Srsf3 fl/fl) conditional allele were crossed with C57BL/6-Tg(Pf-4-icre)Q3Rsko/J mice to generate platelet factor-4 (Pf4)-Cre-Srsf3 KO (MK-LCMΔ/Δ) mice, where deletion of exon 2 and 3 of Srsf3 was under the control of the Pf4 promoter. Pf4-Cre-Srsf3 KO mice were bred with red fluorescent protein (RFP) mice ubiquitously expressing a cytoplasmic pbActin-CMV-DsRed T3 transgene to generate RFP Pf4-Cre-Srsf3 KO mice. Pf4-Cre mice were separately crossed with tomato mice (B6.Cg-Gt(ROSA)26Sortm14(CAG-tdTomato)HzeJ) (Bar Harbor, USA), where Cre excision results in tdTomato expression in Pf4 expressing cells. Mice ubiquitously expressing nuclear green fluorescent protein (NZeg-eGFP) were also used.<br><br>Mice of both sexes between the ages of 6-12 weeks were used. Mice were maintained in a dark/light cycle of 12 hours / 12 hours. Animals were kept at room temperature with a range of 18-24 degrees C. Humidity within the animal facility was between 50-90%. |
| Wild animals | This study did not involve wild animals. |
| Reporting on sex | Mice of both sexes were used in this study. |
| Field-collected samples | This study did not involve samples collected from the field. |
| Ethics oversight | Animal ethics oversight was done by the Monash Animal Ethics Committee (MARP) 3, Monash University, Australia. |

Note that full information on the approval of the study protocol must also be provided in the manuscript.

# Flow Cytometry

## Plots

Confirm that:

☒ The axis labels state the marker and fluorochrome used (e.g. CD4-FITC).

☒ The axis scales are clearly visible. Include numbers along axes only for bottom left plot of group (a 'group' is an analysis of identical markers).

☒ All plots are contour plots with outliers or pseudocolor plots.

☒ A numerical value for number of cells or percentage (with statistics) is provided.

## Methodology

| | |
|---|---|
| Sample preparation | Sample preparation is clearly described in the methods section.<br><br>Mouse bone marrow, spleen and blood, as well as human bone marrow samples were used to assess and/or isolate sub-populations including but not limited to megakaryocytes, platelets and hematopoietic stem cells. Sample may have undergone red cell lysis, density gradient centrifugation and/or lineage depletion before staining (see Methods for more details). |
| Instrument | FACS: Influx, BD Biosciences.<br>Flow cytometry: LSR II, BD Biosciences. |

| | |
|---|---|
| Software | BD FACS Sortware 1.2.0.142 was used for FACS.<br>BD FACSDIVA Software 8.0.1 was used for flow cytometric analysis.<br>FlowJo v10 was used for flow cytometric analysis. |
| Cell population abundance | The abundance of populations are described throughout the text and figures. Following sorting, a small aliquot was re-analysed on the sorter or analyser (eg. HSC), megakarocytes were assessed and counted under the light microscope. |
| Gating strategy | For ease, gating strategies are presented as figures in the manuscript and supplementary information. Defined populations were set based on negative straining of isotypes (eg. HSC); initially, megakarocyte sub-populations were sorted and confirmed to be distinct populations visually under the microscope. |

☐ Tick this box to confirm that a figure exemplifying the gating strategy is provided in the Supplementary Information.
